# Near-Infrared Photobiomodulation of Living Cells, Tubulin, and Microtubules *In Vitro*

**DOI:** 10.3389/fmedt.2022.871196

**Published:** 2022-05-04

**Authors:** Michael Staelens, Elisabetta Di Gregorio, Aarat P. Kalra, Hoa T. Le, Nazanin Hosseinkhah, Mahroo Karimpoor, Lew Lim, Jack A. Tuszyński

**Affiliations:** ^1^Department of Physics, University of Alberta, Edmonton, AB, Canada; ^2^Department of Mechanical and Aerospace Engineering, Politecnico di Torino, Turin, Italy; ^3^Scholes Lab, Department of Chemistry, Princeton University, Princeton, NJ, United States; ^4^Vielight Inc., Toronto, ON, Canada; ^5^Department of Oncology, University of Alberta, Edmonton, AB, Canada

**Keywords:** photobiomodulation, cells, tubulin, microtubules, therapeutic devices, medical technologies, neurodegenerative diseases

## Abstract

We report the results of experimental investigations involving photobiomodulation (PBM) of living cells, tubulin, and microtubules in buffer solutions exposed to near-infrared (NIR) light emitted from an 810 nm LED with a power density of 25 mW/cm^2^ pulsed at a frequency of 10 Hz. In the first group of experiments, we measured changes in the alternating current (AC) ionic conductivity in the 50–100 kHz range of HeLa and U251 cancer cell lines as living cells exposed to PBM for 60 min, and an increased resistance compared to the control cells was observed. In the second group of experiments, we investigated the stability and polymerization of microtubules under exposure to PBM. The protein buffer solution used was a mixture of Britton-Robinson buffer (BRB aka PEM) and microtubule cushion buffer. Exposure of Taxol-stabilized microtubules (~2 μM tubulin) to the LED for 120 min resulted in gradual disassembly of microtubules observed in fluorescence microscopy images. These results were compared to controls where microtubules remained stable. In the third group of experiments, we performed turbidity measurements throughout the tubulin polymerization process to quantify the rate and amount of polymerization for PBM-exposed tubulin vs. unexposed tubulin samples, using tubulin resuspended to final concentrations of ~ 22.7 μM and ~ 45.5 μM in the same buffer solution as before. Compared to the unexposed control samples, absorbance measurement results demonstrated a slower rate and reduced overall amount of polymerization in the less concentrated tubulin samples exposed to PBM for 30 min with the parameters mentioned above. Paradoxically, the opposite effect was observed in the 45.5 μM tubulin samples, demonstrating a remarkable increase in the polymerization rates and total polymer mass achieved after exposure to PBM. These results on the effects of PBM on living cells, tubulin, and microtubules are novel, further validating the modulating effects of PBM and contributing to designing more effective PBM parameters. Finally, potential consequences for the use of PBM in the context of neurodegenerative diseases are discussed.

## 1. Introduction

Photobiomodulation was accidentally discovered in 1967 through experiments performed by Endre Mester that attempted to reproduce previously published results by McGuff et al. (1) on the destruction of cancerous tumors in rats using a ruby laser. In Mester's experiments, which used a laser with a significantly lower power, hair growth (2) and wound healing (3) near tumor sites in mice were observed instead of tumor reduction. In the years to follow, these results were applied to human patients with skin ulcers (4, 5). This treatment came to be known as low-level laser therapy (LLLT) and then eventually low-level light therapy which includes the usage of LEDs instead of lasers. Recently, the term LLLT has been superseded by PBM, which doesn't suffer from the lack of clarity surrounding the definition of “low-level” (6–8). Originally, such therapies were largely focused on the reduction of pain and inflammation or the stimulation of wound healing and tissue growth. To this end, positive results have been obtained in clinical (9) and animal (10, 11) trials. Consequently, PBM therapy (PBMT) has seen significant interest as a form of treatment in sports-related injuries (12–15) and has been studied in connection with sports performance (16).

Recently, a wide range of studies involving the application of PBMT to various neurodegenerative diseases has emerged in the literature (17), demonstrating some remarkable results in both clinical and animal (18) trials. This is typically referred to as brain PBM (19, 20). One common approach to brain PBM involves the usage of transcranial PBM (tPBM), which has demonstrated various positive effects in several different studies. For example, the study performed by dos Santos Cardoso et al. (21), which used a 100 mW transcranial laser with an emission wavelength of 660 nm, found an improved inflammatory response in the brain of aged rats. In a separate study, Xuan et al. found that tPBM with an 810 nm diode laser administered to mice after traumatic brain injury enhanced memory and learning (22). Transcranial PBM has also been studied with regards to major depressive disorder, a systematic review of which can be found in Caldieraro and Cassano (23). In the study by Figueiro et al. (24), the usage of low-level “bluish-white” light as PBMT in Alzheimer's patients and those with related dementia resulted in improved metrics connected to the sleep and mood of treated patients. A model for how light could interact with sleep and circadian rhythms, and the potential effects on mood and cognition are discussed in LeGates et al. (25). A review of PBM as a therapy for Alzheimer's disease is provided in dos Santos Cardoso et al. (26). In a recent proof-of-concept study, significant improvements in clinical signs of Parkinson's disease after treatment with NIR PBM have been found (27). The effect of NIR PBM on brain activity (resting and evoked) in humans using MRI has also been investigated (28). Lastly, a recent study on the supplementation of NIR tPBM with intranasal PBM yielded significant improvements in the cognition of dementia patients (29). These brain PBM studies are relevant to our investigation into the effects on tubulin and microtubules (MTs), which are integral structures of brain cells with rather interesting and unique electrical properties (30).

In terms of effective parameters, it is established that there is a dosage window, below which there is insufficient energy to cause a response, and above which there could be a negative response (known as a biphasic dose response) (31, 32). In oncology, PBM has been reviewed for safety and efficacy (33). It should be noted, however, that there is a significant amount of conflicting literature regarding the effects of PBM on cancer cells (34). For example, Kara et al. found that PBM applied using a 1, 064 nm laser with several power output levels (0.5, 1, 2, and 3 W) induced an increased viability and proliferation in isolated cancer cells that varied with the power (35). On the other hand, Djavid et al. found that application of PBM at 685 nm prior to radiotherapy could inhibit the growth of HeLa cells (36). Ultimately, it appears that there is a strong dependence on the dosimetry and parameters used (37). In the meantime, the effect of PBM on cancer remains in open discussion (38). Our findings here provide additional insights and could help to build a better understanding of the physical mechanisms involved in this field.

In this report, we present results from a series of experiments that examined the effects of PBM on cell lines and cellular components that could help to explain improved brain health with PBM. For these studies, we chose living cancer cells, as well as microtubules and tubulin due to their significant electrostatic properties, in order to examine the effects of PBM. A description of the PBM device used in the experiments, the Vielight Neuro Alpha, is provided in Section 2. We present the first experiments performed in Section 3 which investigated the effects of exposure to PBM delivered by the Vielight device on HeLa and U251 cancer cells lines. In Section 4, we turn to the experiments performed on PBM-exposed microtubules and tubulin. First, we present fluorescence microscopy results obtained from experiments with rhodamine labeled microtubules exposed to PBM delivered by the device. This is followed by turbidity assays of PBM-exposed tubulin which used absorbance measurements performed throughout the polymerization process to track and quantify the polymerization. Finally, our results and subsequent consequences for the use of PBM in the context of pain and neurodegenerative diseases are discussed in Section 5. Concluding remarks and future outlooks are provided in Section 6.

## 2. Experimental Device Information

The device used in the experiments, the Vielight Neuro Alpha 2, is a wearable brain photobiomodulation device developed by Vielight Inc. (39) that delivers transcranial-intranasal brain PBM via a headset and intranasal applicator. Both components of the device are equipped with LEDs that emit 810 nm NIR light pulsed at an oscillation frequency of 10 Hz commensurate with alpha brain waves. Only the intranasal applicator is used in the experiments. The intranasal applicator has one LED which targets the ventral area of the brain via the nasal cavity (method patented by Vielight Inc.). A summary of the Vielight Neuro Alpha device specifications can be found in Table 1.

**Table 1:** Vielight Neuro Alpha parameters.

**Parameter**	**Value (Intranasal)**	**Value (Transcranial)**
Light source	810 nm LED × 1	810 nm LED × 4 (3 posterior, 1 anterior)
LED output power	25 mW	100 mW (posterior) and 75 mW (anterior)
LED pulse frequency	10 Hz	10 Hz
Pulse duty cycle	50%	50%
Beam spot-size	~1 cm^2^	~1 cm^2^
LED power density	25 mW/cm^2^	100 mW/cm^2^ (posterior) and 75 mW/cm^2^ (anterior)
Application time (default)	20 min	20 min
*E*_Net_ delivered (per LED)	15 J	60 J (posterior) and 45 J (anterior)
Net energy dose (per LED)	15 J/cm^2^	60 J/cm^2^ (posterior) and 45 J/cm^2^ (anterior)

The 10 Hz pulse frequency delivered by the Neuro Alpha has been investigated and demonstrated efficacy for studies in dementia (29) and traumatic brain injury (40). This gives us cause to investigate the 10 Hz effect on structures of brain cells, particularly tubulin and microtubules, in search of further understanding of the mechanisms. This pulse rate also correlates with electroencephalogram (EEG) alpha brain wave “entrainment” (41) and cellular light absorption (42). Additionally, the 810 nm wavelength has shown several benefits for mental applications compared to other wavelengths used in PBM, such as 633 nm, 655 nm, etc. (43). Finally, the question of whether or not low-level NIR light can penetrate the skull has been studied in cadaver experiments (43–45) and studies with *ex vivo* skulls (46), as well as *in silico* using Monte Carlo (MC) simulations (47, 48). Together, these studies suggest that a low energy 810 nm light source pulsed at a frequency of 10 Hz can penetrate the skull and could have a potential positive effect on mental health.

## 3. Exposure of Living Cells to PBM *In Vitro*

### 3.1. Experimental Procedures

HeLa cells (human cervical cancer cell line) and U251 cells (human glioblastoma cell line) were obtained from Drs. Gordon Chan and Roseline Godbout at the University of Alberta's Cross Cancer Institute. The HeLa cells were cultured in high glucose Dulbecco's Modified Eagle's Medium (DMEM), 5% [v/v] fetal bovine serum (FBS), and antibiotics (100 U/ml penicillin and 100 μg/ml streptomycin). The U251 cells were maintained in low glucose DMEM, 10% [v/v] FBS, and antibiotics (100 U/ml penicillin and 100 μg/ml streptomycin). The cells were cultured at 37 °C with 5% CO_2_ unless otherwise stated.

**Figure 1:**
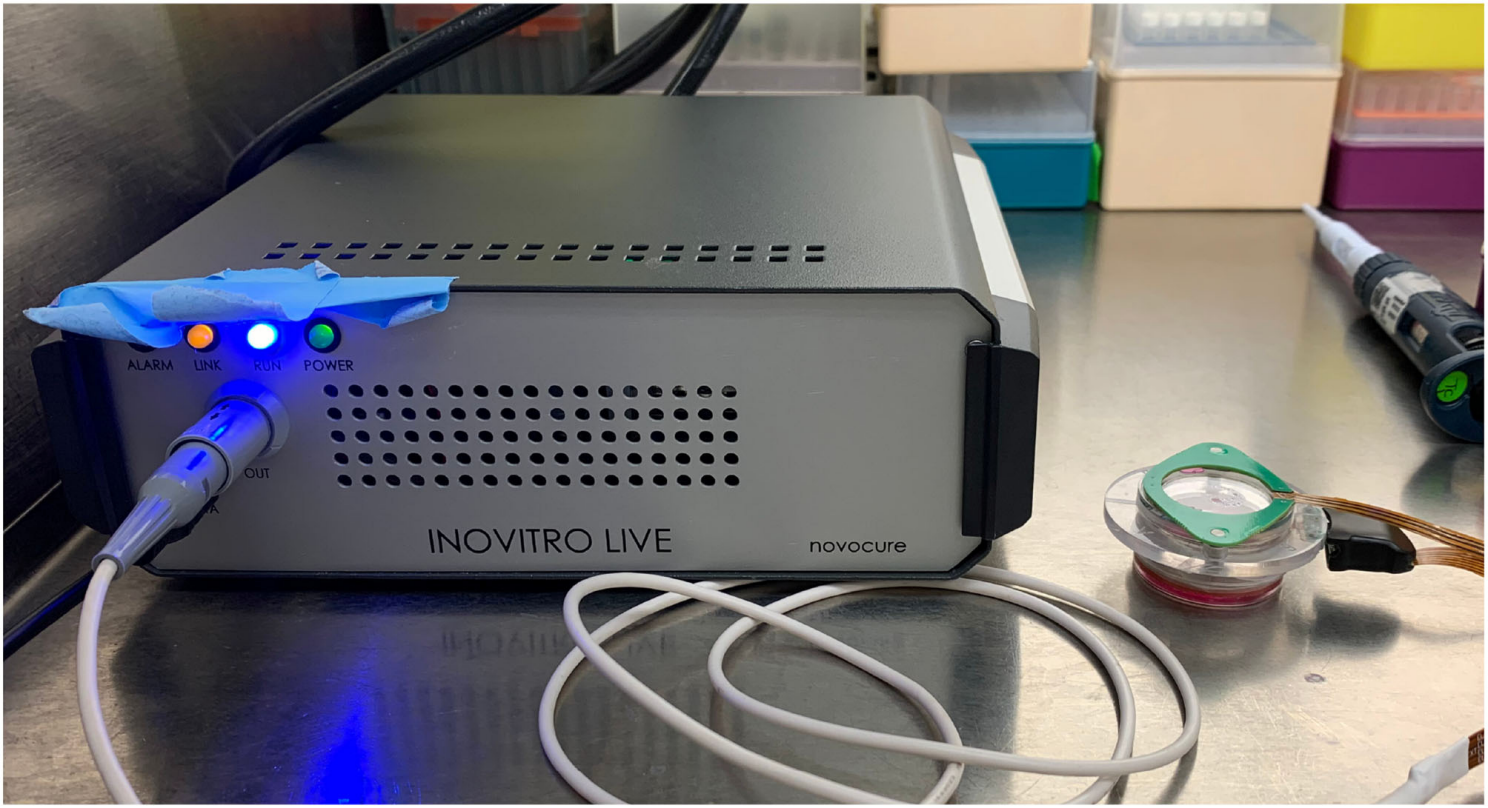
A digital photograph of the inovitro live system. The inovitro live TTFields generator is shown on the left with the ceramic insert and the attached cover heating element visible on the right.

The cells were set up at ~ 60–80% confluence on the day of treatment with PBM and tumor-treating fields (TTFields). Novocure Ltd. (Haifa, Isreal) has developed various systems to study the effects of TTFields on cancer cells in laboratory settings. We used the Novocure inovitro live system (shown in Figure 1) which comprises four major components: a TTFields generator, a high-capacitance ceramic insert with several electrodes, a cover heating element with temperature control, and a laptop computer running the accompanying operation software. The TTFields generated are alternating electrical fields with a low intensity (1–3 V/cm) and an intermediate frequency (50–500 kHz), known to disrupt cancer cell division. The generator is connected to the ceramic cylinder insert containing two perpendicular pairs of electrodes that alternate such that the orientation of the TTFields is rotated 90° every second. The cover heating element attaches to the sockets on the top of the ceramic insert and is held in place magnetically. The operation software for the inovitro live system allows one to set up various parameters including the TTFields frequency (50–500 kHz), target temperature (37 °C, optimal temperature for cultured cells), cover temperature (37–55 °C to prevent condensation to the cell culture lid), and the duty cycle (1 or 2 pairs of electrodes). Additionally, readings of the media temperature, as well as the current and the resistance measured between each pair of electrodes, are recorded every 3 s by the system and stored in the software. The ambient temperature must be lower than the target temperature in order for the TTFields generator to supply the heat required to maintain the target temperature in the culture media; thus, higher intensities will require a lower ambient temperature. The ceramic insert is placed inside a 35 mm cell culture dish. Primarily, the system was used to provide measurements and graphs of the temperature, current, and resistance as a function of time throughout exposures of living cells to the Vielight LED. Therefore, any factors affecting the cells and/or culture media when exposed to PBM (alongside TTFields) would lead to real-time changes in the logged data.

The Vielight Neuro Alpha intranasal LED applicator used as the source of light, was attached to the center of the lid of the cell culture plate. The ceramic insert of the inovitro live device was placed inside the cell culture dish. The dish was placed in an incubator chamber with the temperature set to 23 °C in an atmosphere of 5% CO_2_. TTFields were applied for 1 h in either the absence or presence of exposure to the Vielight LED, and the data logged by the system was acquired by the software. Previous studies reported that the efficacy of TTFields on inhibiting cancer cell proliferation is frequency-dependent, with optimal frequencies leading to the highest reduction in the cell counts (49–54). Thus, we investigated the effect of the Vielight LED applicator alongside two different frequencies of TTFields, 50 and 100 kHz, on two different cancer cell lines, HeLa and U251.

### 3.2. Results for PBM-Exposed Cells

**Figure 2:**
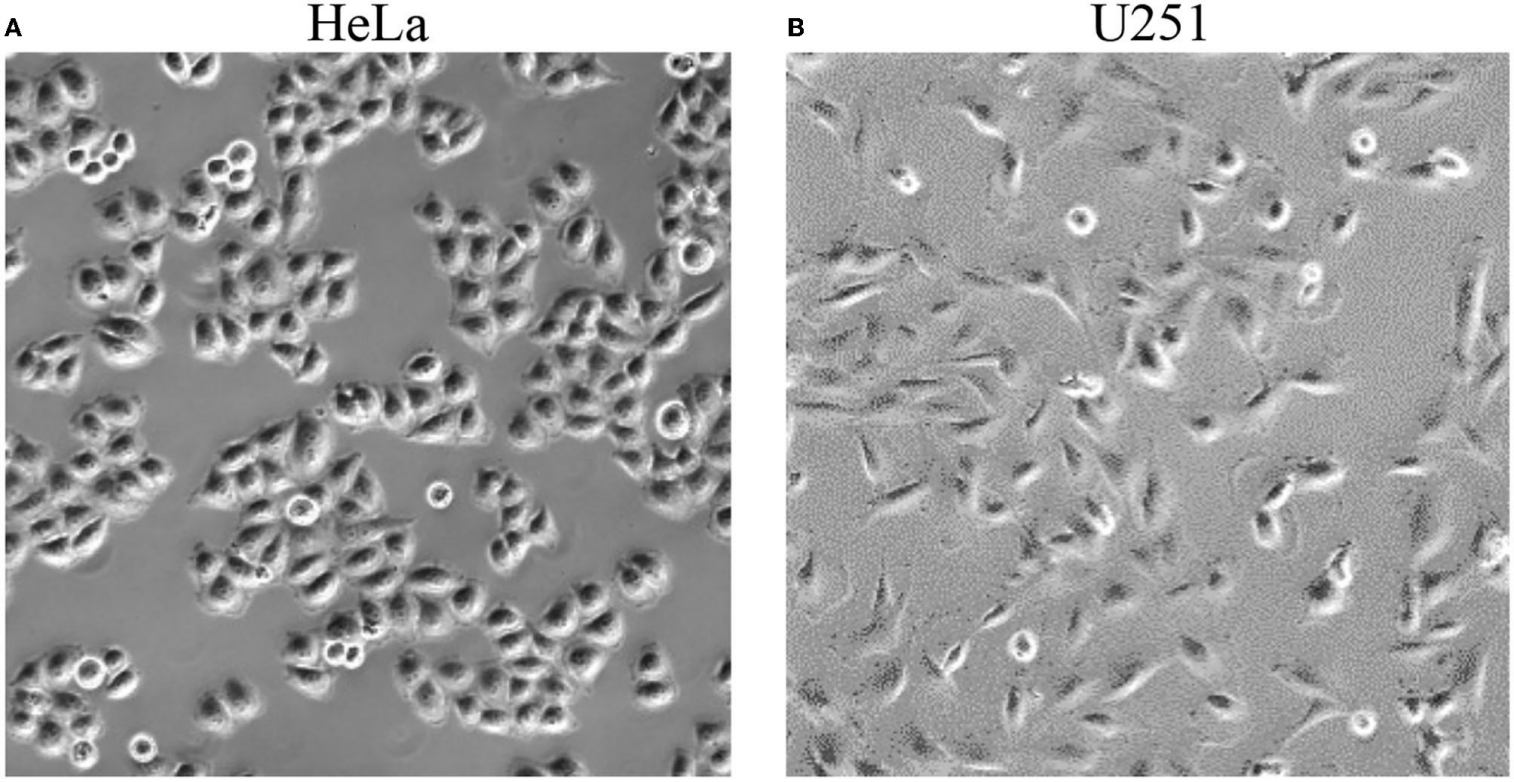
Representative photographs of HeLa (left, **A**) and U251 (right, **B**) cells taken before exposure to TTFields and the Vielight LED.

**Figure 3:**
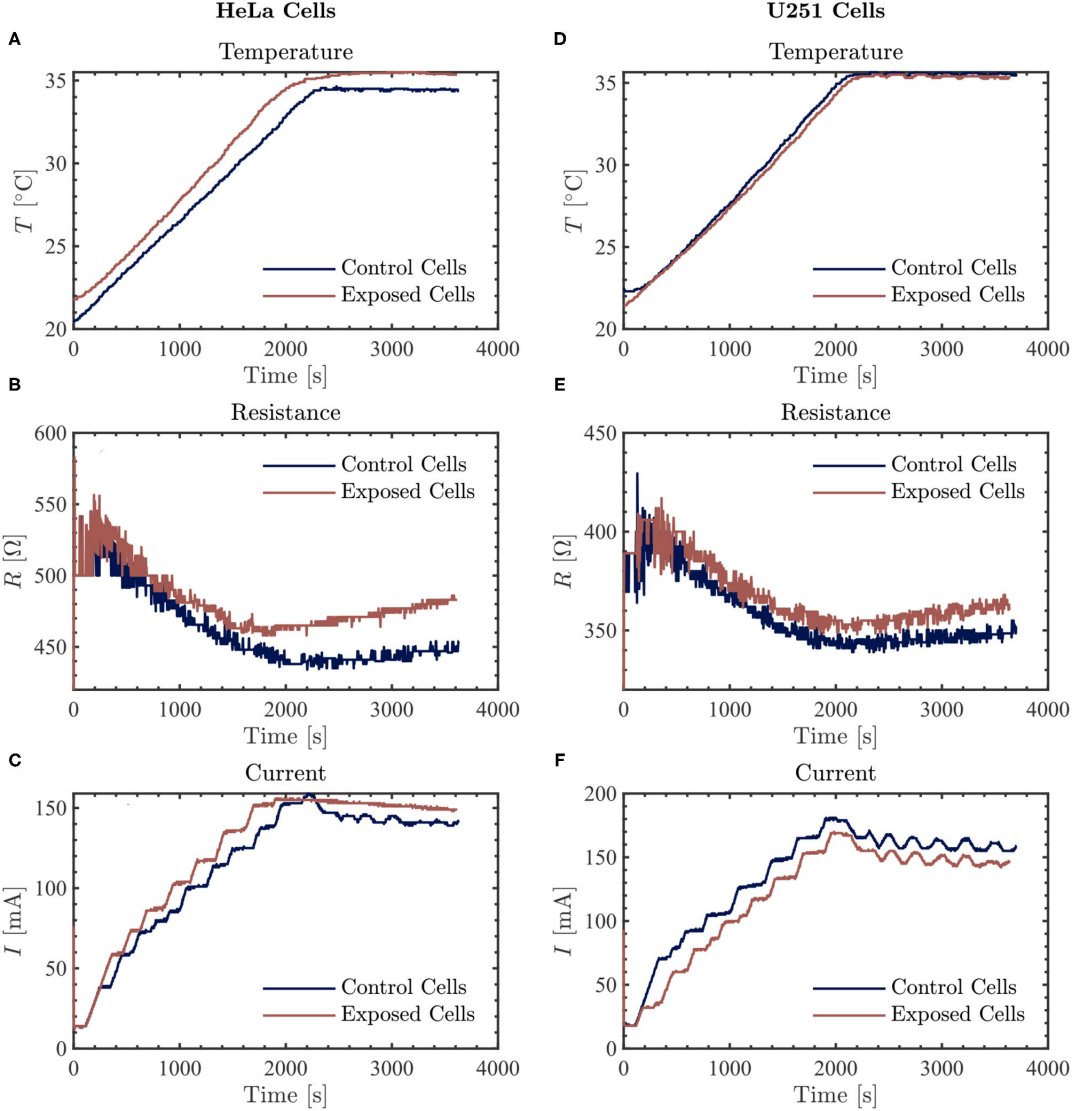
The resulting average temperature (top), resistance (middle), and current (bottom) graphs measured by the inovitro live software for 50 kHz TTFields applied to HeLa (left, **A–C**) and U251 (right, **D–F**) cells in the absence (control) or presence of Vielight LED exposure for 1 h with the software settings as described in the text. The control and Vielight LED-exposed results are shown in blue and red, respectively. Data shown for both cell lines are taken from a single independent experiment out of *N* = 2 (for HeLa) and *N* = 1 (for U251) total replicate experiments performed.

**Figure 4:**
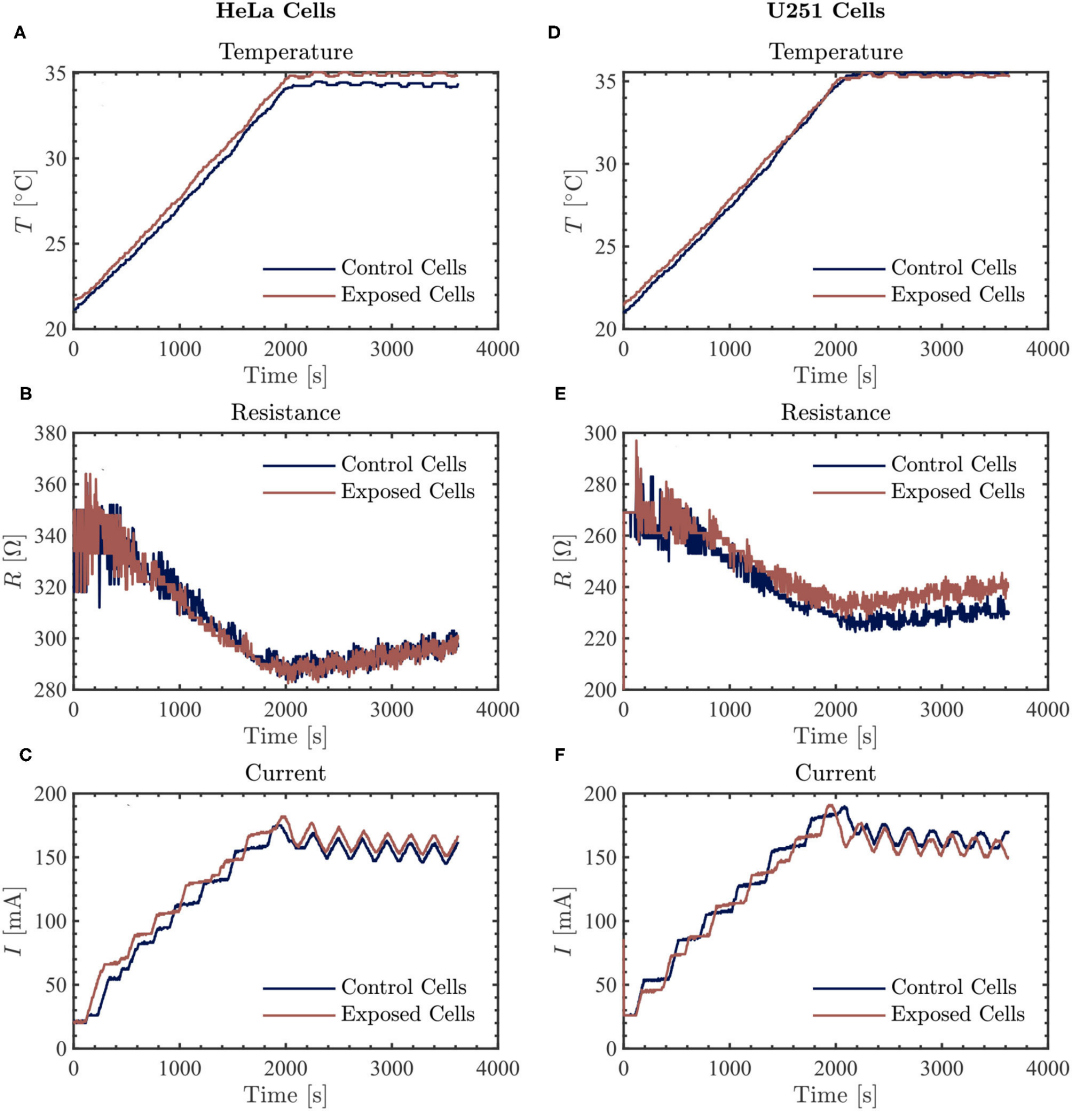
The resulting average temperature (top), resistance (middle), and current (bottom) graphs measured by the inovitro live software for 100 kHz TTFields applied to HeLa (left, **A–C**) and U251 (right, **D–F**) cells in the absence (control) or presence of Vielight LED exposure for 1 h with the software settings as described in the text. The control and Vielight LED-exposed results are shown in blue and red, respectively. Data shown for both cell lines are taken from a single independent experiment out of *N* = 2 total replicate experiments performed.

Prior to treatment using TTFields, with or without simultaneous exposure to PBM included, 3 ml of fresh culture media was added to the cervical cancer and glioblastoma cell lines (this is the minimum volume recommended for use with the inovitro live system). Images of cells were taken before any exposure (Figure 2). There were no changes in cell morphology after treatment with the TTFields and Vielight LED (data not shown). Cells were exposed to the Vielight LED at the same time as TTFields. Control samples that were only treated with TTFields were also included in each scenario. In each experiment, it took ~ 35–40 min for the media to reach ~ 37 °C after which the temperature remained stable (Figures 3A,D, 4A,D). Both the control and the Vielight LED-exposed samples displayed similar trends in the measured temperatures, currents, and resistances. At ~ 37 °C, the measured currents became relatively stable for both tested frequencies (Figures 3C,F, 4C,F). Interestingly, for both cell lines studied in the experiments that used 50 kHz TTFields, the Vielight LED-exposed samples exhibited a significant increase in resistance values that grew with time (after the medium temperature was reached) compared to the controls (Figures 3B,E). Consequently, the most substantial increases in resistance measured were at 1 h: ~ 485 Ω in the exposed HeLa cells compared to ~ 450 Ω in the control (in *N* = 2 independent trials performed), and ~ 365 Ω in the exposed U251 cells compared to ~ 350 Ω in the control (*N* = 1). This corresponds to percent increases of ~ 7.8% and ~ 4.3% in the measured resistances of the exposed HeLa and U251 cells, respectively. As one would expect, this resulted in a lower measured current (reduced by ~ 9.4% at 1 h) in the U251 cells exposed to the Vielight LED, compared to the control (Figure 3F). Surprisingly, however, the current values measured in the Vielight-treated HeLa cells for 50 kHz TTFields maintained a larger value (increased by ~ 7.1% at 1 h) compared to the control samples (Figure 3C), even though their resistance was also increased. Lastly, we note that the measured differences in resistance between the exposed and control HeLa cells might have been more dramatic if the media reached equivalent temperatures (Figure 3A); the reduced media temperature of the control samples by ~ 1 °C has the effect of increasing the resistance, thereby reducing the measured differences. Consequently, this increased resistance in the control cells reduces the measured current, which could also help to partially explain the peculiar increase in the current observed in the (more resistant) PBM-exposed HeLa cells.

At the frequency of 100 kHz, we did not observe a consistently significant difference in the measured current values between the control and the Vielight LED-treated samples in both HeLa (*N* = 2) and U251 (*N* = 2) cells (Figures 4C,F). Furthermore, the resistance values measured in the Vielight LED-treated HeLa cells were essentially the same as the control samples (Figure 4B). This is contrary to the previously discussed scenario involving 50 kHz TTFields, in which the HeLa cells exhibited the most significant increase in resistance after exposure to the Vielight LED. However, in the case of the Vielight LED-treated U251 cells, the observed effects on the resistance were consistent with the previous scenario, resulting in an increased resistance measured compared to the control cells (Figure 4E). In particular, for the largest increase in resistance measured compared to the control during the 1 h exposure time, the exposed U251 cells reached ~ 240 Ω while the control was ~ 230 Ω (an increase of ~ 4.3%, roughly identical to before). These results suggest that the effect of the Vielight LED on cells might be frequency-dependent, with this dependence also varying between cell lines.

## 4. Exposure of Microtubules and Tubulin to PBM *In Vitro*

Our studies of the potential effect of the Vielight Neuro Alpha PBM device on microtubules and tubulin *in vitro* began with an investigation using fluorescence microscopy. In particular, we reconstituted rhodamine labeled tubulin samples and polymerized microtubules from the samples, half of which were kept as control samples while the remainder were exposed to the LED intranasal applicator of the Vielight device for 2 h. The samples were then viewed under a Zeiss Axio Examiner.Z1 fluorescence microscope using a red fluorescent protein (RFP) filter set and the results were imaged with a Hamamatsu C9100 electron-multiplying charge-coupled device (EMCCD) camera. These experiments were followed by studies of tubulin exposed to PBM delivered by the Vielight LED, which was then allowed to polymerize while turbidity (absorbance) measurements were performed throughout the process to monitor and quantify the results.

### 4.1. Fluorescence Microscopy Results

We reconstituted rhodamine labeled tubulin (TL590M, stock lyophilized powder obtained from Cytoskeleton, Inc.) by first resuspending it in ice-cold G-PEM buffer to a final tubulin concentration of 4 mg/ml (≃ 36.4 μM). The G-PEM buffer was prepared with guanosine triphosphate (GTP—stock concentration of 100 mM) added to cold PEM buffer (aka BRB80—80 mM PIPES *p*H 6.9, 0.5 mM EGTA, 2 mM MgCl_2_) to a final GTP concentration of 1 mM. Specifically, we combined 5 μl of GTP with 495 μl of cold PEM buffer to assemble the G-PEM buffer, and immediately placed it on ice. The required number of labeled tubulin aliquots were removed from storage at 4 °C thereafter and placed on ice. The cold G-PEM buffer, as well as microtubule cushion buffer/tubulin glycerol buffer (MTCB—BRB80 diluted in 60% [v/v] glycerol), were both added to each labeled tubulin aliquot at a 4:1 ratio (4 μl of G-PEM was added to each aliquot followed by 1 μl of MTCB). Finally, the aliquots were mixed well using a variable speed vortex mixer. Each aliquot of labeled tubulin prepared in this fashion was then snap frozen in LN_2_ and stored at −80 °C for future use.

**Figure 5:**
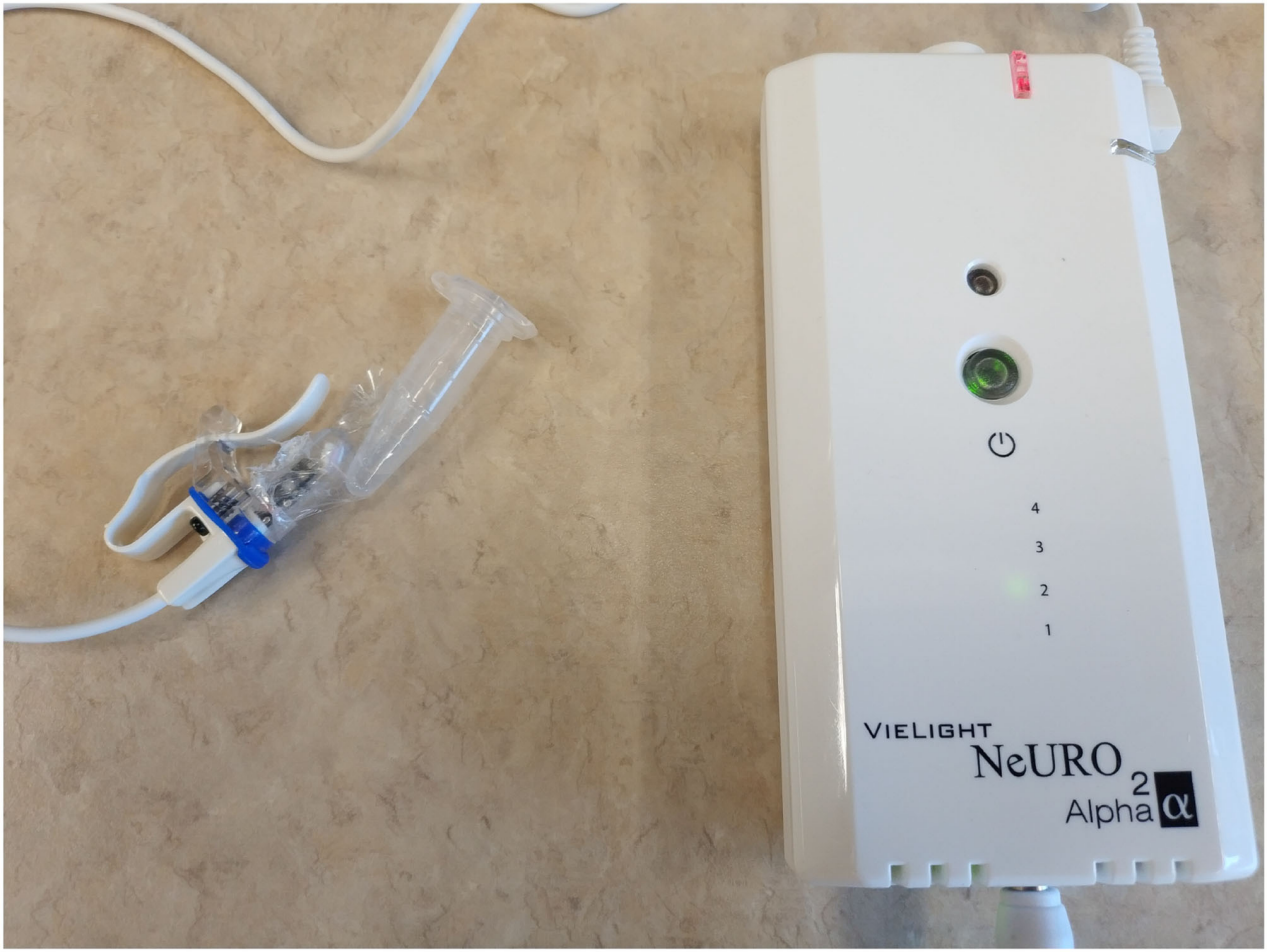
A digital photograph of a sample aliquot of tubulin solution attached to the LED intranasal applicator of the Vielight Neuro Alpha device for PBM exposure.

The experiments began by removing the required number of labeled tubulin aliquots from the freezer and immediately placing them into a 37 °C water bath to polymerize for 30–45 min. During the polymerization process, a solution of PEM-T [PEM buffer + paclitaxel (as Taxol)—TXD01, lyophilized powder obtained from Cytoskeleton, Inc. and resuspended in dimethyl sulfoxide (DMSO)] was prepared simultaneously that was used to stabilize the microtubules after removing them from the water bath. Finally, we attached one of the sample aliquots to the LED intranasal applicator of the Vielight Neuro Alpha device (see Figure 5), covered the setup, and performed an exposure (at room temperature) for 6 of the preset cycles corresponding to a total exposure time of 120 min. The concentration of Taxol present in the PEM-T solution was treated as a variable, and several strengths of solution (2 μM, 4 μM, and 20 μM Taxol) were explored in the various experiments.

**Figure 6:**
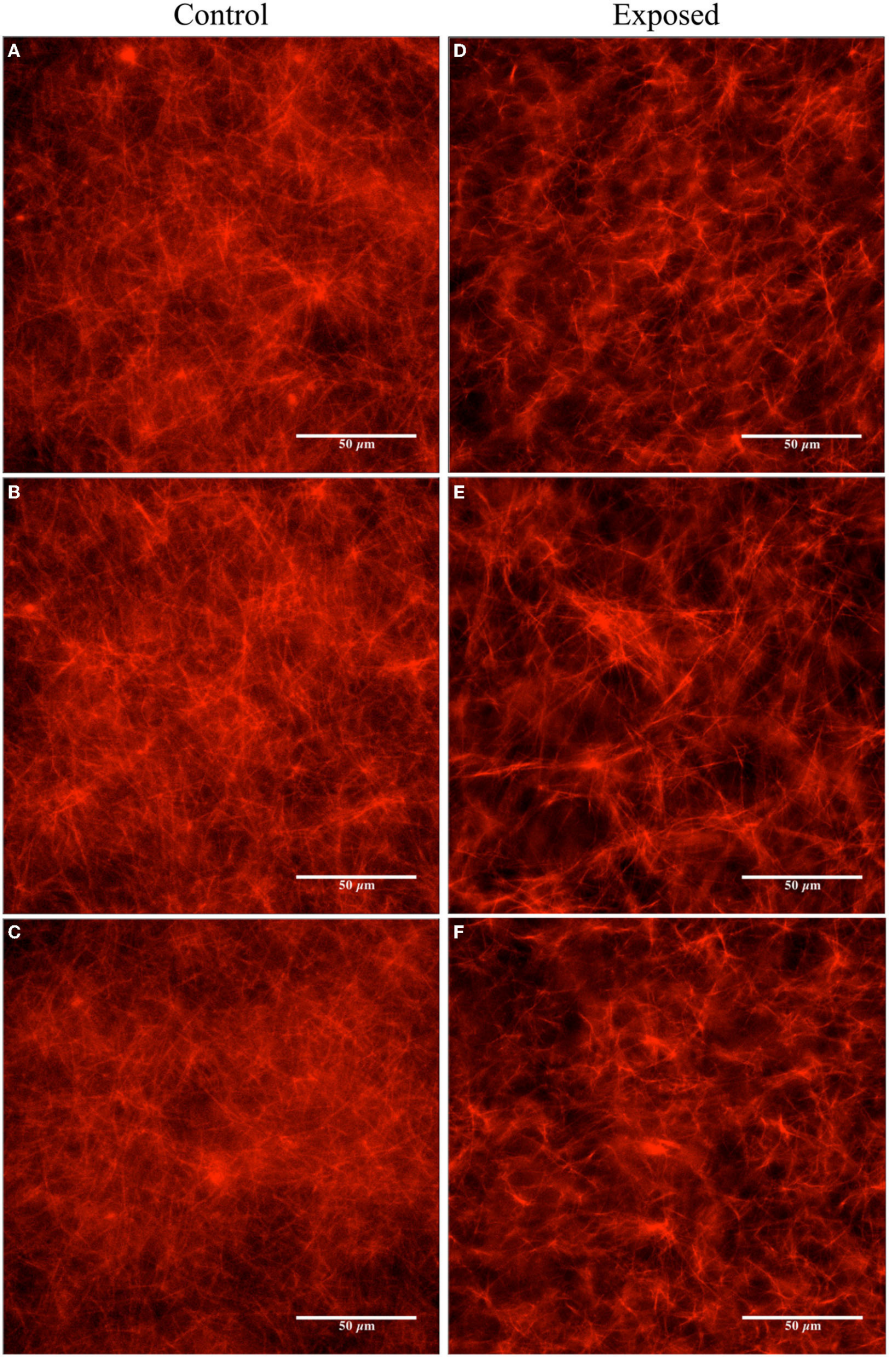
Fluorescence microscopy images obtained for the unexposed and the Vielight LED-exposed MTs (labeled with rhodamine) stabilized in PEM-T (20 μM Taxol and ~ 2 μM tubulin). The control group is shown on the left **(A–C)** and the exposed (2 h) on the right **(D–F)**. Images are representative of *N* = 1 independent replicate experiments.

**Figure 7:**
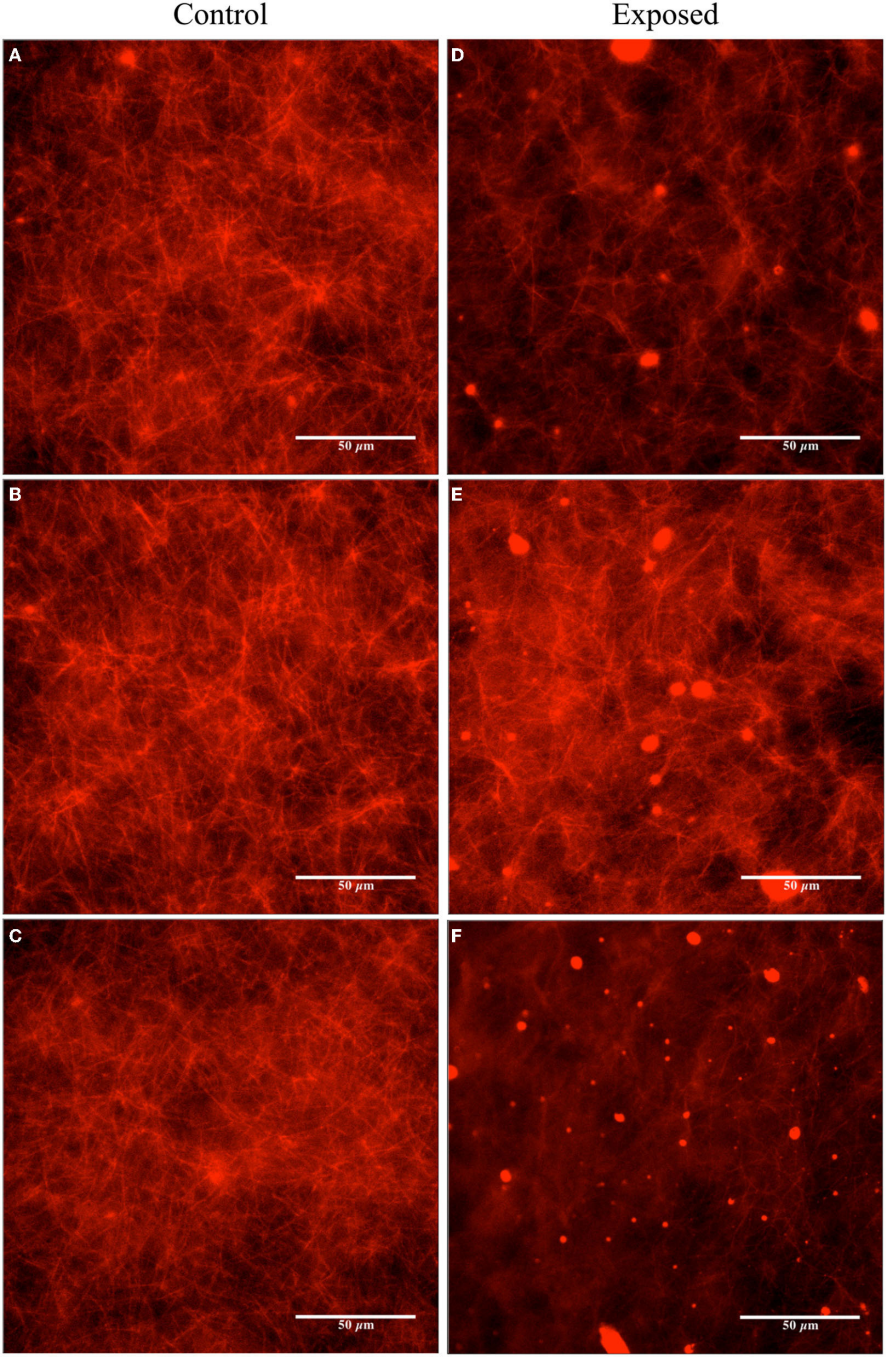
Fluorescence microscopy images obtained for the unexposed and the Vielight LED-exposed MTs (labeled with rhodamine) stabilized in PEM-T (4 μM Taxol and ~ 2 μM tubulin). The control group is shown on the left **(A–C)** and the exposed (2 h) on the right **(D–F)**. Images are representative of *N* = 3 independent replicate experiments.

In the first trials, we experimented with a high concentration of Taxol (20 μM) in the PEM-T solution, 100 μl of which was added to both the control and pre-exposed tubulin samples after polymerization in the 37 °C water bath (yielding a final tubulin concentration of ~ 2 μM). Fluorescence microscopy was performed on the control sample at the beginning of the exposure (images not shown) and on both samples after the 120 min exposure was complete. Images of the control samples showed no changes after 2 h of resting at room temperature. In the exposed sample, a slight reduction in the overall amount/concentration of microtubules present was observed compared to the control sample after 2 h of exposure to the Vielight LED, as shown in the fluorescence microscopy images presented in Figure 6. In the subsequent trial, a fresh set of tubulin samples were polymerized and stabilized in PEM-T, however this time using a 90% reduction in the concentration of Taxol present in the buffer solution added to each sample. After performing the 2 h exposure to the Vielight LED, the unexposed control sample, which had been resting at room temperature for this period, showed significant signs of degradation as the MTs were unstable and depolymerizing. Consequently, no conclusions were drawn from the control vs. exposed samples in this trial. Finally, an intermediate concentration of Taxol was used (4 μM), and remarkably, a significant effect was observed in the Vielight LED-exposed MT sample. In particular, we observed a large reduction in the concentration of microtubules as well as the formation of aggregates of tubulin, as shown in the fluorescence microscopy images provided in Figure 7. The latter trial was replicated twice, finding a similar effect of exposure to the Vielight LED leading to microtubule destabilization and a reduction in the total polymer mass. These findings suggest that the effect of PBM on Taxol-stabilized MTs, with the specific parameters used by the Vielight Neuro Alpha, is potent enough to counteract the strong MT-stabilizing effects of Taxol (at 4 μM).

Image analyses were performed using the fluorescence microscopy images presented in Figures 6, 7 to quantify the observed differences. The details and full results of these analyses are available in the Supplementary Material. Here, we present the average pixel brightness values calculated for the red channel for each of the sets of three control and exposed images presented in Figures 6, 7A–C and Figures 6, 7D–F, respectively. For the fluorescence microscopy images obtained for MTs stabilized with a high Taxol concentration (Figure 6), we obtained mean values of 124.1 ± 34.77 for the control and 102.1 ± 37.83 for the Vielight LED-exposed images. In the scenario that used an intermediate Taxol concentration, a lower mean value of 95.4 ± 33.18 was obtained for the exposed images, as expected. This supports our observation that the PBM-exposed MTs in the latter scenario achieved a significantly lower total polymer mass. It should be noted, however, that this method results in rather substantial uncertainties due to the large variance in red brightness values between all of the pixels in each fluorescence microscopy image obtained. To obtain a more reliable quantification of the observed effect, we turn to turbidity measurements.

### 4.2. Turbidity Measurement Results

In order to further quantify the observed reduction in polymerization discovered in the previously presented results, we performed turbidity measurements on tubulin samples exposed to the Vielight LED applicator; the polymerization of tubulin in solution causes the media to become turbid, and consequently, the amount of light scattered off the sample (measured as optical density, OD) will increase with turbidity as measured by the microplate reader system. Thus, turbidity measurements can be used as a proxy to measure tubulin polymerization. Similar to the previously discussed experiments, we began by reconstituting lyophilized tubulin in G-PEM buffer. In these experiments, we used porcine brain derived ultra-pure unlabeled tubulin (>99%) obtained from Cytoskeleton, Inc. (T240) resuspended in a mixture of G-PEM buffer (1 mM final concentration of GTP) and microtubule cushion buffer at a 9:1 ratio (10% MTCB final concentration). Final tubulin concentrations of 2.5 mg/ml and 5 mg/ml (≃ 22.7 μM and ≃ 45.5 μM) were used in the experiments to follow.

Initially, baseline absorbance measurements of tubulin turbidity without any exposures to the Vielight LED were performed to validate that the turbidity protocol, measurements, and equipment were working correctly and produced results consistent with expectations based on the literature. Absorbance measurements performed on tubulin samples with Taxol or CCI-001 added were also included as additional validations/sanity checks (control wells containing only the buffers, and blank wells, were also measured—data not shown here). The former is a chemotherapy drug known to enhance tubulin polymerization and stabilize microtubules (as mentioned previously) while the latter is a colchicine-derived cytotoxic compound known to have an opposite effect that prevents the polymerization of β-tubulin. The administration of each drug was performed by adding it to the G-PEM buffer used in the reconstitution of unlabeled tubulin aliquots. Hereafter, we refer to these compounds as G-PEM-T and G-PEM-C, for the Taxol and CCI-001 containing buffers (with GTP), respectively. In both cases, we used stock solutions of the drug suspended in DMSO at initial concentrations of 2 mM. The CCI-001 was obtained locally from the University of Alberta's Department of Oncology. The G-PEM-T and G-PEM-C were prepared by first assembling the G-PEM as before, and then by adding 1 μl of the corresponding drug to the G-PEM buffer, yielding final concentrations of ~ 4 μM. Upon reconstitution of the unlabeled tubulin (to a concentration of 22.7 μM) using either drug-containing buffer alongside MTCB (9:1 ratio), final drug concentrations of ~ 3.6 μM were obtained.

**Figure 8:**
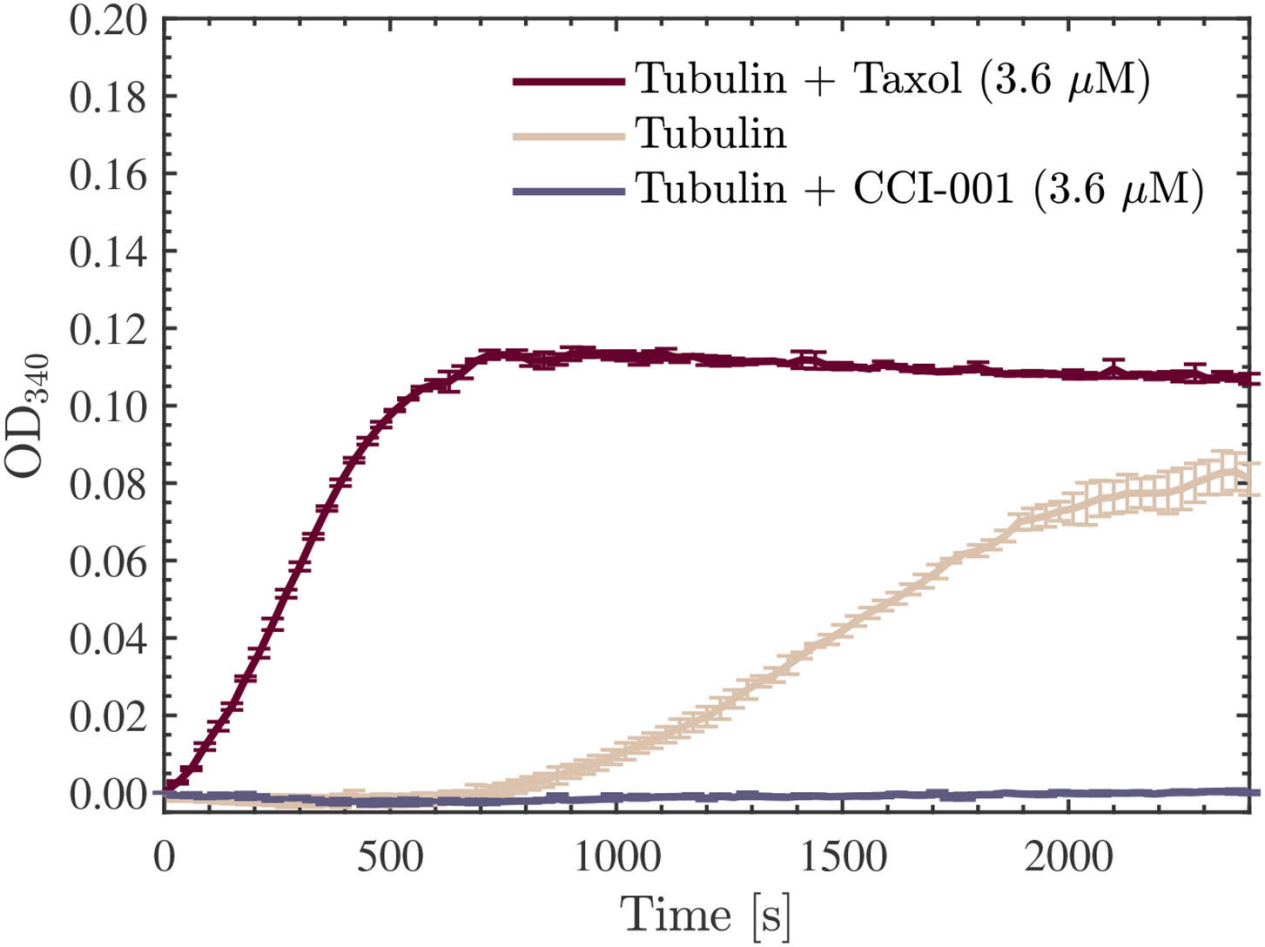
Average optical density results obtained for absorbance measurements performed at 340 nm, every 30 s for 40 min, using a SpectraMax iD5 microplate reader. The red, beige, and purple curves presented are for the 22.7 μM tubulin samples measured in all three scenarios discussed: Taxol-stabilized (3.6 μM), drug-free, and CCI-001 (3.6 μM), respectively. The data presented was collected in *N* = 1 independent replicate experiments.

As the samples were being prepared for this first series of turbidity measurements, the microplate reader was warmed to 37 °C. Absorbance measurements at 340 nm were performed every 30 s for 40 min using a SpectraMax iD5 Multi-Mode Microplate Reader from Molecular Devices (see Table 2 for the full list of settings and parameters used in our measurements). The data was collected directly by the accompanying data-acquisition software, SoftMax^®^ Pro 7.1 (55). Three separate wells, each containing ~ 100 μl of solution, were measured for each scenario. Measurements of the optical density at 340 nm (OD_340_) averaged over the 3 wells in each scenario are provided in Figure 8 as a function of time. As expected, a sigmoidal population growth curve with a distinct nucleation phase was obtained for the ordinary tubulin samples, while an enhancement and negation of the growth were observed in the tubulin samples reconstituted with G-PEM-T and G-PEM-C, respectively. Moreover, we note that the nucleation phase was absent in the Taxol-containing tubulin samples—a well-known effect documented in the literature.

**Table 2:** Full details of the settings used in our tubulin turbidity measurement protocol based on absorbance readings.

**Parameter**	**Value**
*t*_total_	2400 s
*t*_int_	30 s
*N*_reads_	81
Plate type	96 well standard (clear bottom)
Well height/depth	14.6 mm
λ_abs_	340 nm
Shake before	Yes, 5 s orbital, medium
Shake between	Yes, 5 s orbital, medium

**Figure 9:**
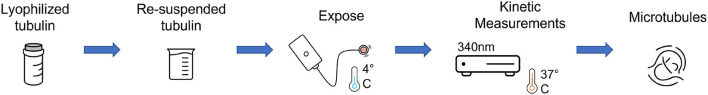
A schematic of the experimental procedure used for turbidity measurements of the Vielight LED-exposed tubulin samples.

We proceeded with the first experiment involving turbidity measurements performed on tubulin samples exposed to the Vielight LED. Upon reconstitution of the unlabeled tubulin, samples of 100 μl were aliquoted, and prepared for exposure to the Vielight LED. Triplicate control and exposed samples were produced for use in the first set of turbidity measurements performed. Additionally, as we did not want any of the tubulin samples to polymerize during the exposures (or while awaiting exposure), both the control and exposed samples were kept at 4 °C in the fridge throughout this process. A schematic of the experimental procedure is provided in Figure 9.

The first set of turbidity experiments were performed on tubulin solutions with a concentration of 22.7 μM. This value was chosen because it is close to the maximum physiological value of 24 μM found in living cells (56). Three separate exposures to the Vielight LED (one per exposed sample) were performed at 4 °C for 30 min each. As we used only one LED, the control samples, as well as those pre- or post-exposure, were kept in the same fridge that the exposures were performed in during this time. After 90 min, once all three separate exposures were completed, the samples were immediately pipetted into a 96 well microplate. We placed 100 μl of each sample into the wells, labeled the microplate grid, and began absorbance measurements at 340 nm using the microplate reader (pre-heated to 37 °C). Interestingly, we observed a significant reduction in the OD_340_ rates and final values measured in the samples exposed to the Vielight LED, suggesting a lower total amount of polymer mass achieved compared to the control samples. This is consistent with our observations in Section 4.1 of a reduction in polymerized MTs after exposure to the Vielight LED.

**Figure 10:**
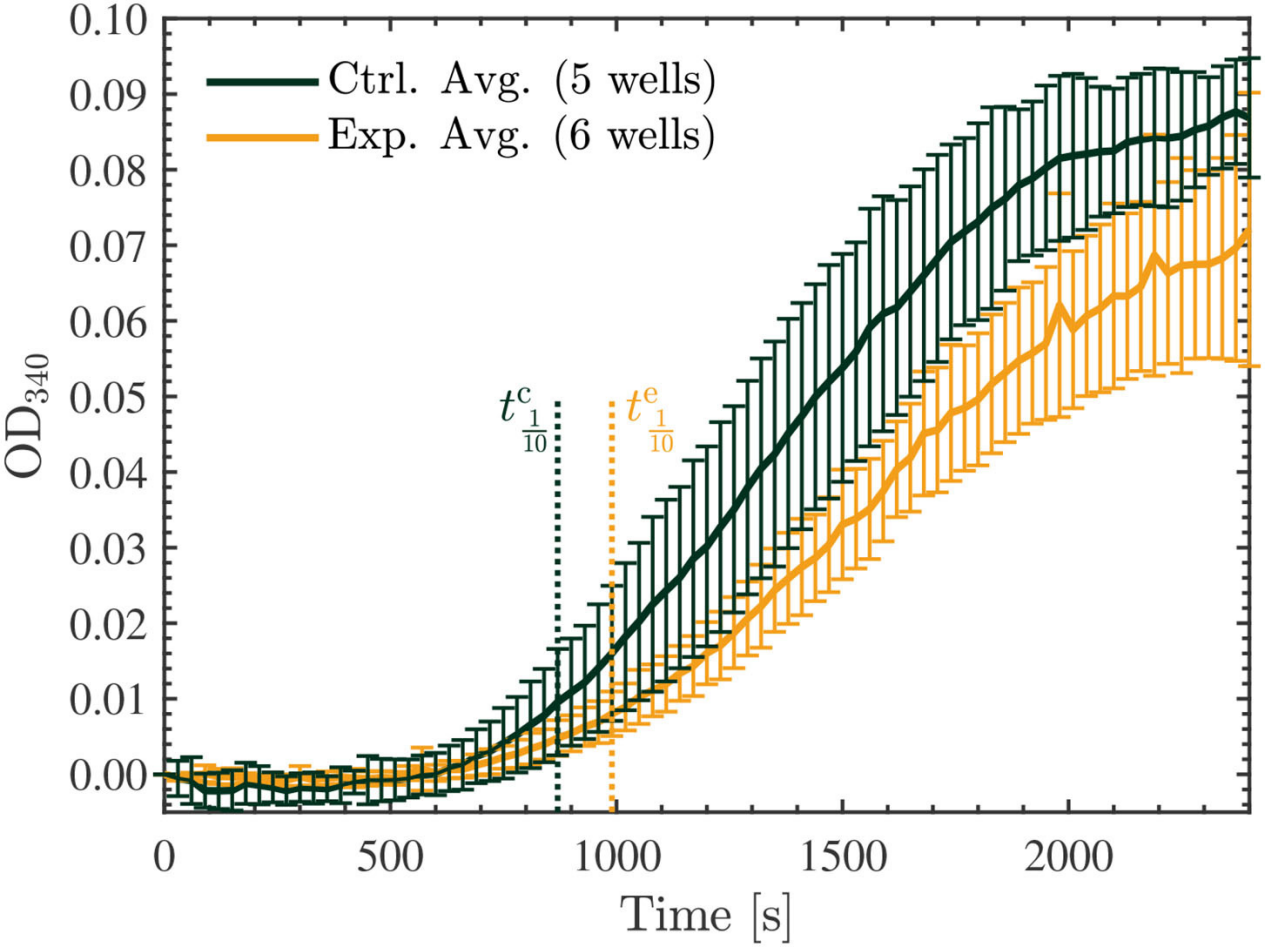
Average optical density results of absorbance measurements performed at 340 nm, every 30 s for 40 min, on the 22.7 μM control (green) and exposed (yellow) tubulin samples. The tenth times of $t_{1/10}^{c}≃870$ s and $t_{1/10}^{e}≃990$ s calculated for the control and exposed curves, respectively, are overlayed as the dotted vertical lines shown. The data presented was collected in *N* = 2 independent replicate experiments.

This experiment was repeated using another 5 samples (3 Vielight LED-exposed and 2 controls) and the same consistent results and overall trends were obtained. The results of both experiments were combined and the average curves for the control and exposed samples obtained have been provided in Figure 10 along with their standard deviations. Further analyses of the data were carried out using OriginPro^®^ 2021b (v9.85) (57). First, sigmoidal Boltzmann fits were applied to both the exposed and control curves using the nonlinear curve fitting tool in OriginPro^®^. Second, the maximal slope values (*V*_max_)—which represent the maximal growth rates achieved during the polymerization reactions—were calculated directly, including their uncertainties, for each of the fits. These were calculated to be $V_{\text{max}}^{c}≃5.0\pm 0.1$ mOD/min and $V_{\text{max}}^{e}≃3.8\pm 0.1$ mOD/min for the control and exposed curves, respectively. Lastly, based on the values obtained in the sigmoidal curve fitting, the time required to produce 10% of the maximal value of polymer, called the tenth time (*t*_1/10_) (58), was calculated for each curve as well. These results were $t_{1/10}^{c}≃870$ s and $t_{1/10}^{e}≃990$ s for the control and exposed turbidity measurements, respectively. These values were overlayed onto the turbidity curves, as shown in Figure 10. The full details and results of these additional analyses can be found in the Supplementary Material. Notably, we found that the maximal slope and tenth time for the average exposed curve were significantly smaller and longer, respectively, compared to those calculated for the (average) control curve.

**Figure 11:**
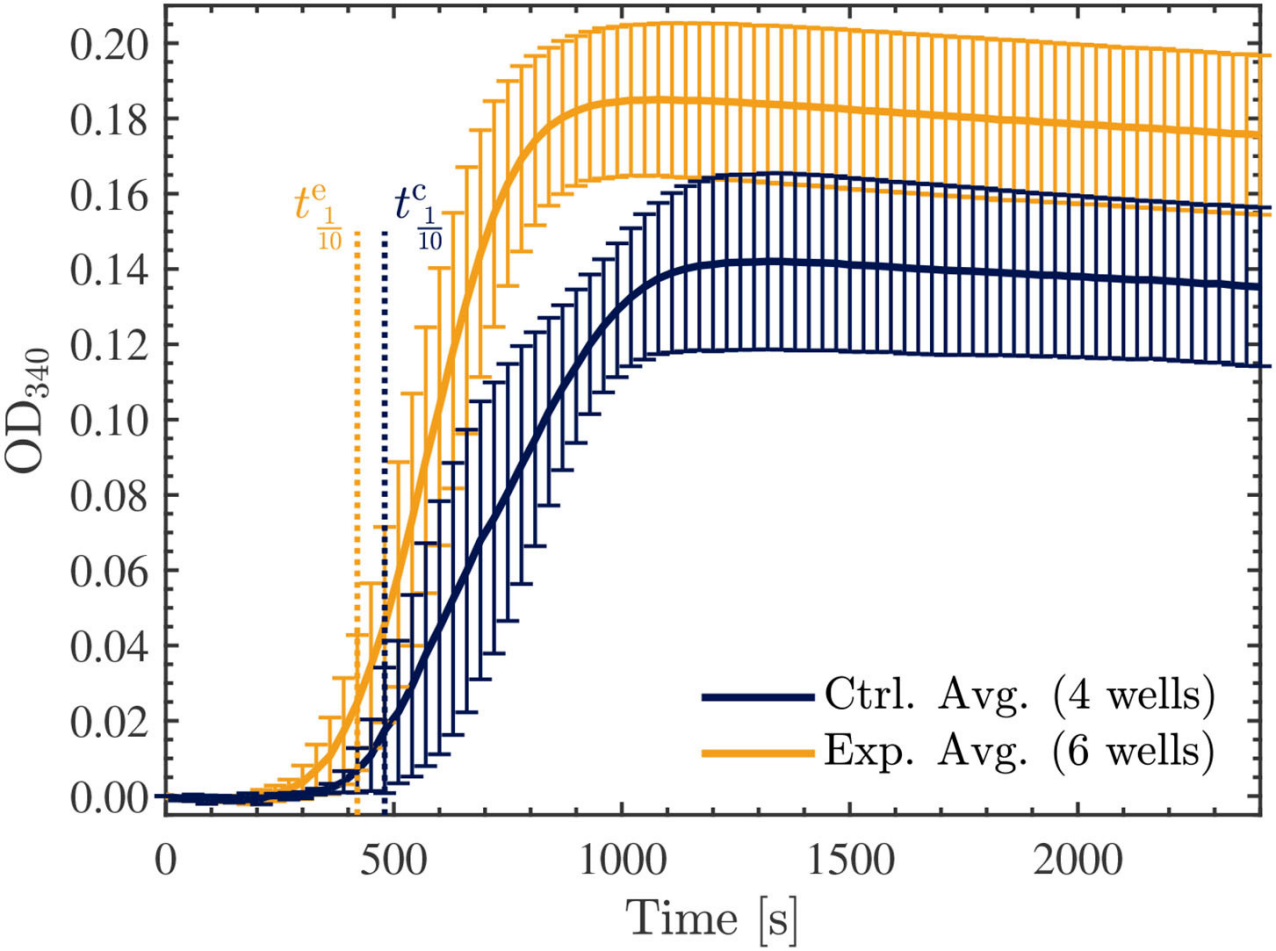
Average optical density results of absorbance measurements performed at 340 nm, every 30 s for 40 min, on the 45.5 μM control (blue) and exposed (yellow) tubulin samples. The tenth times of $t_{1/10}^{c}≃480$ s and $t_{1/10}^{e}≃420$ s calculated for the control and exposed curves, respectively, are overlayed as the dotted vertical lines shown. The data presented was collected in *N* = 2 independent replicate experiments.

Finally, we repeated another series of two experiments, each involving 3 exposed and 2 control samples as before, however, in these trials we investigated the effect of the Vielight LED on the samples with double the concentration of tubulin, at 45.5 μM. Surprisingly, in both experiments performed, the opposite effect was observed compared to the lower concentration samples. Specifically, the Vielight LED-exposed samples exhibited faster polymerization rates and achieved a greater total polymer mass, as shown in Figure 11. This effect was consistent between all six exposed samples and the control wells in their corresponding experiments. As before, the maximal slopes were calculated directly from the sigmoidal fits, yielding $V_{\text{max}}^{c}≃17.6\pm 0.5$ mOD/min and $V_{\text{max}}^{e}≃33.2\pm 0.8$ mOD/min for the control and exposed curves, respectively. The calculated tenth times were $t_{1/10}^{c}≃480$ s and $t_{1/10}^{e}≃420$ s. As mentioned, and based on the slopes and tenth times calculated, the opposite effect was observed in the Vielight LED-exposed samples when using double the concentration of tubulin. As one would expect though, the tenth times calculated for both the control and exposed 45.5 μM tubulin samples were substantially shorter than those calculated in the 22.7 μM scenarios. Remarkably, the maximal growth rate of the Vielight LED-exposed 45.5 μM tubulin samples was nearly double that of the control group. In the work of Tuszynski et al., which performed several tubulin turbidity assays (using 27.3 μM tubulin) in the presence of two different MT-stabilizing drugs, notable increases in the *V*_max_ of this order were observed, but only due to the presence of 10 μM paclitaxel (an increase from 13 to 21 mOD/min) or 100 μM lankacidin C (an increase from 13 to 35 mOD/min) (59). Additionally, there were essentially no nucleation phases present in their data for these scenarios (as expected). This implies that the effect induced on the 45.5 μM tubulin samples by exposure to the Vielight LED, although delayed (as nucleation phases were still present), is exceptionally strong and nearly equivalent to that of potent MT-stabilizing drugs.

## 5. Discussion

While the field of PBM continues to receive validation for its many applications through various safety and efficacy studies, investigations into its mechanisms and cellular effects need to also grow in tandem. Through over 50 years of research history, while a credible amount of work has accumulated in the understanding of mitochondrial activity, gene transcription, physiological modulations, etc., there is a considerable gap in knowledge, particularly regarding cellular mechanisms. In the context of this report, we sought to understand how a PBM device such as the Vielight Neuro Alpha may have produced the efficacy as reported in various clinical studies.

The first set of experiments involved the use of HeLa and U251 cell lines to explore whether there was any effect on living cells. We found a significant effect in both cell lines exposed to the Vielight LED during application of 50 kHz TTFields—in increasing resistance to current flow, which is counter-intuitive for clinical efficacy. Simultaneously, the current flow measured in the exposed HeLa cells was greater than that in the control samples. These seemingly dichotomous effects were in play at the same time.

In PBM literature, authors have suggested that neurodegenerative diseases could be due to excitotoxicity. The excitotoxicity has been attributed to the lack of inhibition on glutamatergic activities, causing intramitochondrial overload of Ca^2+^ resulting in apoptosis (60) and presumably neuronal atrophy in neurodegeneration. They have suggested that PBM inhibits this manner of excitotoxicity buildup, which helps to reduce neurodegeneration. Work on animal models have pointed to the upregulation of glutamate transporter-1 (GLT-1) to inhibit glutamatergic excesses (61). Curiously, with the PBM of living cells in our experiments, inhibition was observed, represented by an increased resistance to the flow of current. This was observed for both HeLa and U251 cell lines exposed to NIR PBM alongside 50 kHz TTFields, whereas at 100 kHz only the exposed U251 cells still exhibited an increased resistance compared to the control cells. In the meantime, the current flow was also increased compared to the control in the case of the HeLa cells exposed to PBM and 50 kHz TTFields (Figure 3C). These findings propose that PBM upregulates the dynamics in cell activities. The increases in excitatory activities are not left unchecked but accompanied by inhibitory activities to maintain a healthy balance or homeostasis. While we observed a significant difference from the control groups at 50 kHz, we did not observe as significant of a difference at 100 kHz, suggesting that outcomes are dependent on current flow frequency, but we have yet to understand why that is the case.

In the second set of experiments, we investigated the stability of microtubules and the polymerization rates of tubulin under exposure to NIR PBM pulsing at 10 Hz. Exposure of Taxol-stabilized microtubules for 120 min resulted in gradual depolymerization and disassembly of the microtubules. This is in contrast to earlier work that have used much higher intensity lasers. In 2007, Chow et al. observed the development of “varicosities” under the exposure to a 830 nm NIR low-level laser (62). They hypothesized that these structures contribute to blocking fast axonal flow and hence reducing nociceptor pain signaling. This was somewhat replicated by Holanda et al. with 808 nm lasers (63). In both cases, they used far higher power to drive the lasers to achieve fast axonal flow blockage: 1 W at 4.5 cm from the microtubule samples in the case of Chow et al., and a 300 mW/cm^2^ intensity in the case of Holanda et al. We only applied a 25 mW/cm^2^ intensity with incoherent NIR light from an LED. With the lower energy intensity, we observed the opposite effect of reduced density in microtubule samples indicating depolymerization and disassembly. This implies that low-intensity PBM does not cause varicosities that block axonal signaling, and we can hypothesize that it could be upregulating neuronal signaling instead. This would be consistent with the body of PBM literature that suggest low-intensity PBM upregulates mitochondrial potential and improves physiological functions. It would be a factor contributing to the improved outcomes in brain functions that are impaired due to trauma or neurodegeneration.

In the context of pain signaling, we can summarize that high-intensity NIR irradiation would form varicosities which slow axonal flow and probably reduce mitochondrial membrane potential (MMP). Low-intensity NIR would probably not reduce nociceptor pain signaling conduction, but high-intensity NIR would. In a similar vein, low-intensity PBM would likely increase MMP, whereas high-intensity does the opposite of reducing MMP.

In the third set of experiments, we performed turbidity measurements throughout the tubulin polymerization process to quantify the rate and amount of polymerization for exposed vs. unexposed tubulin samples at two different concentrations, 22.7 μM and 45.5 μM. The choice of a tubulin concentration of 22.7 μM is representative of the physiological value of living cells. Absorbance measurement results demonstrated a slower rate and reduced overall amount of polymerization in these tubulin samples exposed to low-intensity NIR PBM for 30 min, compared to the unexposed control samples. In line with the microscopy observations in the second set of experiments, we observed a significant reduction in the total polymer mass in the low-intensity PBM-exposed samples. In the higher concentration tubulin samples (45.5 μM) that were studied, we noted the opposite effect; a remarkable increase in the polymerization rates and a greater total polymer mass were achieved in the PBM-exposed samples, commensurate with the usage of strong MT-stabilizing drugs such as paclitaxel and lankacidin C. Connecting this result back to the fluorescence microscopy results, where Taxol-stabilized microtubules were destabilized by exposure to NIR PBM supplied by the Vielight Neuro Alpha device, it appears that the corresponding effect is similar in potency to these drugs.

**Figure 12:**
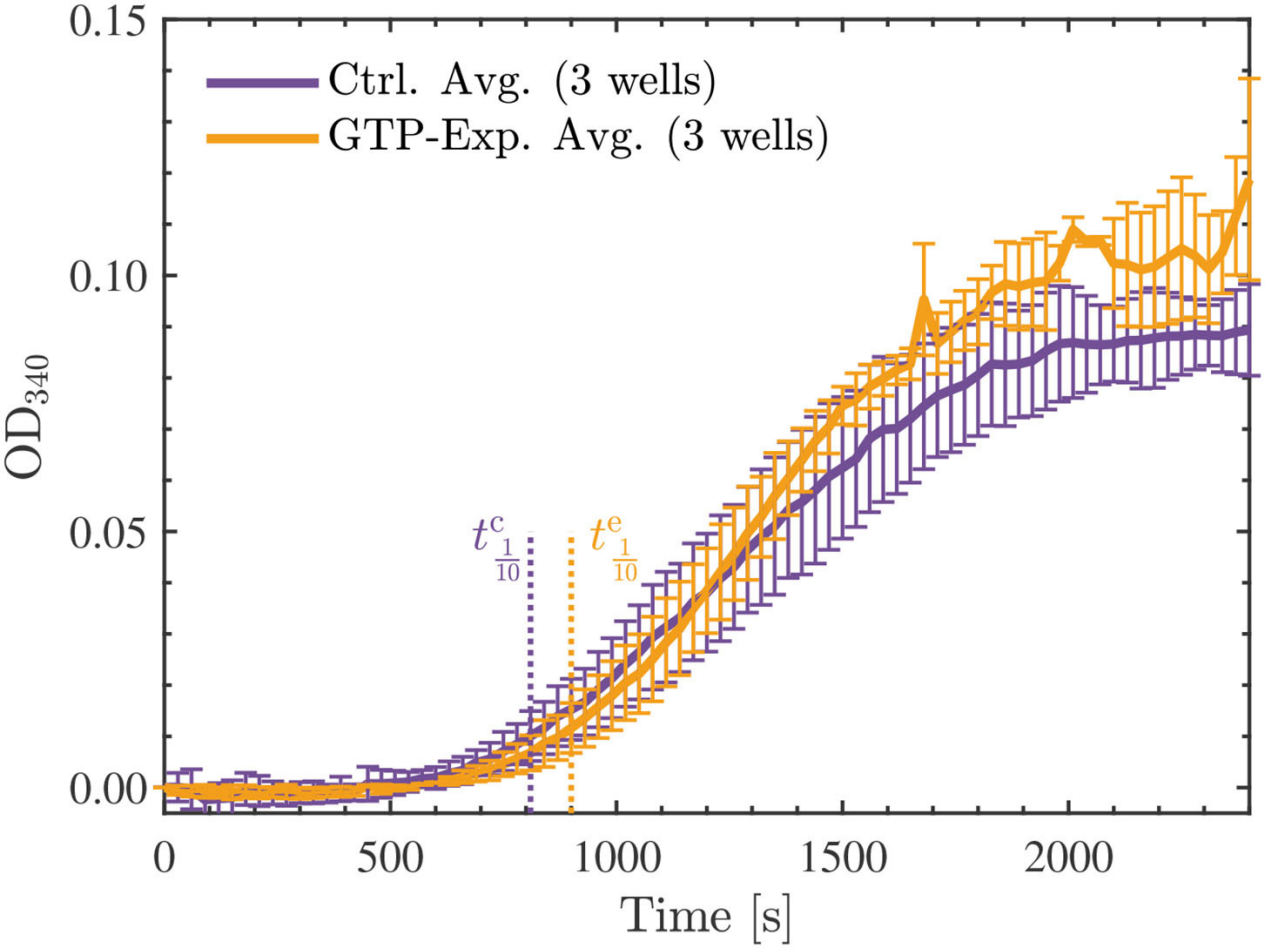
Average optical density results of absorbance measurements performed at 340 nm, every 30 s for 40 min, on the 22.7 μM tubulin samples reconstituted by the standard procedure (control—purple) or with GTP exposed to the Vielight LED (yellow). The GTP exposure was performed for 30 min at 4 °C using 10 μl of stock GTP solution (100 mM) suspended in DI water. The tenth times of $t_{1/10}^{c}≃810$ s and $t_{1/10}^{e}≃900$ s calculated for the control and exposed curves, respectively, are overlayed as the dotted vertical lines shown. The data presented was collected in *N* = 1 independent replicate experiments.

Initially, we hypothesized that the observed reduction in turbidity/polymerization noted for the 22.7 μM tubulin samples resuspended in a solution containing G-PEM buffer and MTCB, and exposed to the Vielight LED, could be a result of interactions between the NIR photons emitted and GTP. These interactions could also potentially explain the depolymerization observed in the fluorescence microscopy experiments presented in Section 4.1; if the GTP cap of polymerized MTs was affected by the exposure then the polymerization could be hindered or catastrophe could be induced. In the case of the exposed tubulin in solution, if the energy were sufficient to hydrolyze GTP to GDP, then the subsequent attempts at polymerization would be negatively affected. Additionally, this would affect the nucleation phase in particular, and should manifest in the turbidity results as a significant increase in the tenth time(s) calculated for the tubulin samples reconstituted using G-PEM buffer assembled with GTP exposed to the Vielight LED (64). To this end, we performed an additional experiment that involved exposing a 10 μl aliquot of stock GTP solution (100 mM) suspended in DI water to the Vielight LED for 30 min at 4 °C. A fresh solution of G-PEM buffer was then prepared using this exposed GTP, and tubulin samples were reconstituted to a concentration of 22.7 μM using the previously described procedure. Turbidity measurements were performed exactly as before, using 3 separate control and exposed samples. The results obtained from the microplate reader were averaged (Figure 12), sigmoidal fits were applied, and the maximal slopes and tenth times were calculated (see Supplementary Material). The tenth times of $t_{1/10}^{c}≃810$ s and $t_{1/10}^{e}≃900$ s calculated were consistent with a delayed nucleation phase present in the samples that used GTP exposed to the Vielight LED. However, if we compare with the tenth time calculated for the control group in the first turbidity experiments performed on 22.7 μM tubulin, a value of $t_{1/10}^{c}≃870$ s, then this apparent delay is significantly shorter. Furthermore, the turbidity curve for the exposed GTP reached a slightly larger maximal value than the control curve. This is contrary to what one would expect, as any loss of GTP in the system should hamper the polymerization. Lastly, taking into account the standard deviations, the control and experimental curves do not differ significantly with any statistical certainty. For these reasons, we refute this initial hypothesis.

The results obtained regarding the turbidity of tubulin samples exposed to the Vielight LED, although paradoxical, do suggest that tubulin and its polymerization processes, in particular, are being affected by the exposure. Furthermore, the opposing nature of these results, which depend on the concentration of tubulin exposed, suggests that the mechanism of interaction producing such effects is a complicated multifactorial problem, likely involving competing processes. We present the following hypothesis regarding such a mechanism, which is largely based on the Smoluchowski equation (65) that describes the simultaneous coagulation of particles involved in processes such as polymerization. A simplified version of this equation can be stated by considering a closed system of volume *V* containing two diffusing spheres with radii of *R*_1_ and *R*_2_. Over time, the spheres will diffuse and coagulate at a rate proportional to the coagulation kernel, *K*, based on the following equation,


(1) $$\begin{array}{r} K=\frac{k}{V}=\frac{4\pi \left(D_{1}+D_{2}\right)\left(R_{1}+R_{2}\right)}{V}, \end{array}$$

where *k* is the macroscopic reaction rate, and *D*_1,2_ are the Einstein diffusion coefficients of the two individual spheres (66). Directly applying this simplified model to our studies, one can consider the two spheres to be tubulin dimers with radii *R*_1_ = *R*_2_ interacting in a solution with a diffusion coefficient *D* ( = *D*_1_ = *D*_2_). Additionally, the joint effective radius σ defined to be the sum of *R*_1_ and *R*_2_—which describes the center-to-center distance necessary between molecules for contact to occur—should also be considered. When the inter-dimer distance is less than σ, then the dimers can interact and aggregate at a rate defined by *K*. Furthermore, the diffusion coefficients, which are proportional to several other parameters, are governed by the following equation,


(2) $$\begin{array}{r} D=\frac{k_{\text{B}}T}{f}=\frac{k_{\text{B}}T}{6\pi R\eta }, \end{array}$$

where *k*_B_ is the Boltzmann constant, *T* is the absolute temperature, *f* is the coefficient of friction, *R* is the radius of the (diffusing) sphere, and η is the solvent viscosity (67). We propose that NIR PBM exposure could have an effect on *R* (and effectively, σ) as well as η, leading to a consequential effect on the diffusion coefficient *D*.

We also emphasize that our samples are solubilized in a solution of different buffers; therefore, they will interact with the adjacent solute, creating what is known as a ‘hydration shell’ in their immediate vicinity (68). According to terahertz absorption spectra studies of protein solutes (validated against molecular dynamics simulations), the dynamic hydration shell around proteins can extend from ~ 14–22 Å, corresponding to at least five layers of water molecules (69). This ability for proteins to induce the structuring of their interfacial water layer (IWL) has been shown to play a critical role in the biological functions of proteins such as folding, enzymatic reactions, and protein-protein interactions (67, 70). There is evidence that red-to-near-infrared (R–NIR) photons, and presumably, other wavelengths (for which bulk water is practically transparent) interact with the bound water, i.e., interfacial water layers. The interaction has at least two biologically significant effects: increased IWL density (volume expansion) and decreased IWL viscosity (71). These findings point to an increase in the radius of the sphere, while the *D*-dependence is more complicated to address. As previously mentioned, *D* is influenced by both *R* and η. While *R* increases upon irradiation, η decreases; therefore, *D* variation depends on the amount with which these parameters are varied. Ultimately, we believe that *D* decreases, making it more difficult for the tubulin dimers to move within the solution. Furthermore, it has been reported that IR exposure of proteins increases interfacial water H-bond cooperativeness and strength, as well as enhances structuring of the hydration shell, which protects proteins against non-specific aggregation in solution, favoring periodic self-assembly (67). Therefore, we hypothesize that a more structured hydration shell could implicate less mobility, which translates into a lower value of *D*.

Additionally, we consider the work of Pollack et al., which has shown that in comparatively large regions (of the order of 100s of μm) around the IWLs surrounding hydrophilic surfaces, water acquires properties that are apparently different from the bulk water. In particular, it was found that the near-surface water at the interface forms an ordered zone with a local charge gradient that excludes solutes at macroscopic scales, which Pollack has called exclusion-zone (EZ) water (72). Based on a follow-up study with nuclear magnetic resonance (NMR) and light absorption spectra, Pollack hypothesized that EZ water exists in a different phase than the bulk water (73). In a further study, Pollack et al. presented contentious evidence that the EZ was negatively charged (74, 75). The effects of incident radiant energy from UV–IR were also investigated and found to induce considerable growth of the EZ in a reversible and wavelength-dependent manner (76, 77). These results also implied that incident IR light may provide the driving force for the charge separation observed in the EZ (77). Although explanations of the underlying mechanisms behind EZs are still in dispute and further study is needed, the presence of this physical phenomenon has been independently replicated and appears to be a genuine effect (78). If true, these results have important implications in our work. The outer surface of tubulin heterodimers is mostly negatively charged, with a particularly large amount of negative charge carried by the C-termini tails (79). Under NIR irradiation, an expanded EZ forms in the surrounding vicinity. This substantial region of negative charge draws in positive counter-ions around the tubulin that concentrate between adjacent like-charged heterodimers, resulting in an attraction that encourages their coalescence (essentially, Feynman's “like-likes-like principle”). This effect could help explain the enhanced polymerization of tubulin observed in the turbidity experiments performed with 45.5 μM tubulin exposed to NIR PBM.

To summarize, under irradiation with the Vielight LED, σ could effectively increase, akin to popcorn kernels swelling up when exposed to microwaves (to some extent). Simultaneously, the diffusivity could be negatively impacted (causing a decrease in *D*), thereby making it more difficult for the tubulin dimers to coalesce. In the case of the lower concentration tubulin samples, where the dimers are less tightly packed (separated by ~ 40% more distance compared to the higher concentration samples), significant diffusion may be necessary for polymerization to occur, which could be hindered by the exposure to the Vielight LED. On the other hand, in the experiments that used less dilute tubulin samples, it could be the case that the exposed dimers (considering their joint effective radius) are already sufficiently tightly packed in the solution, thereby requiring a minimal amount of diffusion for polymerization to occur and their expanded radii can help facilitate their coagulation. Meanwhile, the formation of a sizeable EZ due to NIR irradiation would lead to an additional effect that helps the tubulin polymerize, which together might explain the enhanced polymerization rates observed in the 45.5 μM tubulin samples. This hypothesis will be investigated in future work, using dynamic light scattering (DLS) to probe the effective radii of the irradiated tubulin dimers. There is also a strong possibility that exposure to the Vielight LED is affecting the tubulin and microtubules at a molecular and/or structural level (for example, H-bonds could be affected, or even secondary structures). Raman spectroscopy analyses to probe such effects in PBM-exposed tubulin and microtubules will be the subject of a future publication.

Finally, although the turbidity results presented here for the 22.7 μM tubulin samples are within the range of the physiological concentration of tubulin present in living cells, there are still some significant differences between the environment replicated in our *in vitro* studies and the cellular environment. In particular, microtubules do not exist alone, but rather within a complex network of proteins and ions distributed throughout the cytoplasm. In future studies, we plan to study the effects of PBM on tubulin and MTs in the presence of these additional factors: an increased ionic concentration by including ions such as Na^+^, Ca^2+^, and K^+^; a more viscous environment, through the inclusion of more glycerol; and the decoration of tubulin with microtubule-associated proteins (MAPs), such as tau proteins. The effects of different wavelengths and pulse frequencies used in the PBM treatment will also be explored in future work.

## 6. Conclusion

The three sets of experiments in this report have produced a new understanding of the way that living cells, cellular structures, and components such as microtubules and tubulin respond to low-intensity NIR PBM. They shed additional light on the efficacy of a brain PBM device such as the Vielight Neuro Alpha by providing evidence of non-trivial effects at the cellular and sub-cellular levels.

In the experiments performed with living cells, we demonstrated that HeLa cells exposed to low-intensity PBM (alongside 50 kHz TTFields) respond with increased but controlled flow-through of electrical current. The increased current flow delivered is balanced by an elevated resistance. This shows that PBM balances excitatory stimulation with inhibition, indicating that PBM may reduce excitotoxicity which is relevant to the maintenance of a healthy brain. This effect was no longer present when HeLa cells were exposed to low-intensity PBM accompanied by 100 kHz TTFields, which suggests a strong frequency-dependence of the corresponding effects. Furthermore, we demonstrated that this effect varies between cell lines; when U251 cells exposed to low-intensity PBM and TTFields were studied, the measured resistance increased by a similar order at both tested frequencies of TTFields. As one would expect, this resulted in a simultaneous decrease in the measured current, but it was only significant in the scenario with 50 kHz TTFields.

The second set of experiments deployed fluorescence microscopy to observe the behavior of microtubule structures in response to low-intensity PBM. Microtubules are ubiquitous, integral to neuronal integrity, implicated in signaling, and yet largely unexplored in PBM research. We observed widespread depolymerization and disassembly when Taxol-stabilized microtubules (4 μM Taxol) were exposed to low-intensity NIR light supplied by the Vielight device for 2 h. This is counter-intuitive to expectations consistent with efficacy. Earlier work by other researchers reported high-intensity NIR lasers caused varicosities (swelling) in microtubules that block nociceptor pain signaling, yet in the meantime reduced MMP. Therefore, the low-intensity incoherent LED used in our experiment would likely give the opposite outcomes—depolymerization, increased signaling, and higher MMP. This is consistent with the efficacy of PBM when using low-intensity NIR light sources. Based on these results, our experiments suggest that low-intensity NIR PBM efficacy is associated with the depolymerization of microtubules. Depolymerization of microtubules should also be presented as a reduction in turbidity if the microtubules and the tubulin components are in a stable solution that closely represents the actual environment of live cells. This was the case observed in our final set of experiments performed on PBM-exposed samples with a similar tubulin concentration to that in living cells. However, when we experimented with double this concentration of tubulin exposed to low-intensity PBM, we observed the opposite effect of enhancing tubulin polymerization, suggesting that the underlying processes are likely complex and in competition. In any case, the observed effects of low-intensity PBM on tubulin and MTs were remarkable, displaying a similar potency to the effects of drugs such as paclitaxel and lankacidin C.

While in the literature there are well-documented physiological outcomes of PBM reported, such as improved mental function, the underlying modulation of molecular structures and cellular interactions are far more complex and interesting. The observed behaviors reported in this work regarding cells and cellular structures exposed to low-intensity PBM prompted us to introduce mechanisms that involve tubulin particle diffusion and the restructuring of interfacial water layers. Clearly, more work needs to be done to gain a better understanding of these effects of PBM on human physiology. Ultimately, this improved knowledge can only enhance the precision of personalizing PBM parameters for better outcomes in the future.

## Data Availability Statement

The original contributions presented in the study are included in the article/Supplementary Material, further inquiries can be directed to the corresponding author.

## Author Contributions

The manuscript was written by MS, with additional discussions provided by EDG and LL. JT, EDG, NH, MK, and LL provided detailed edits and feedback. HL performed the experiments with living cells. AK and MS performed the fluorescence microscopy experiments; the images presented were obtained by AK and analyzed by MS. EDG and MS performed the turbidity experiments and calculated the maximal slopes and their uncertainties. EDG applied the sigmoidal fits and computed the tenth times. MS generated all of the final plots using Matlab^®^ R2021b (v9.11.0). JT and the Vielight team (LL, NH, and MK) devised the experiments and provided guidance and support. All authors discussed the results and aided in their interpretation.

## Funding

This study received funding from Vielight Inc., as part of an ongoing collaborative research arrangement where its employees actively assisted in the study. However, final decisions on the study design, data collection and analysis, interpretation of data, the writing of this article, and the decision to submit it for publication were made by JT, MS, EDG, AK, and HL. Additional funding was provided by the National Sciences and Engineering Research Council of Canada (NSERC) Discovery Grant Number RGPIN-2018-03837.

## Conflict of Interest

LL, NH, and MK are employed by Vielight Inc. This study received funding from Vielight Inc. The funder had the following involvement with the study: they provided the device along with the specifications and contributed to the setup and usage of the device. They also contributed to the literature review, information, discussion, and other statements with regards to photobiomodulation (PBM) and the PBM devices in the manuscript. The remaining authors declare that the research was conducted in the absence of any commercial or financial relationships that could be construed as a potential conflict of interest.

## Publisher's Note

All claims expressed in this article are solely those of the authors and do not necessarily represent those of their affiliated organizations, or those of the publisher, the editors and the reviewers. Any product that may be evaluated in this article, or claim that may be made by its manufacturer, is not guaranteed or endorsed by the publisher.

## Supplementary Material

Supplementary Material is available for this article, containing the specific details and results regarding the fluorescence microscopy image analyses, the sigmoidal fits applied to the turbidity data, and their subsequent analyses to obtain the maximal slope values and tenth times.
