# Supplementary material for: Near-Infrared Photobiomodulation of Living Cells, Tubulin, and Microtubules In Vitro
Source: Front Med Technol. 2022 May 4;4:871196. doi: 10.3389/fmedt.2022.871196 (PMC9115106; doi:10.3389/fmedt.2022.871196)
Supplement: Supplementary file 1 [file Data_Sheet_1.pdf]

frontiers

Supplementary Material

1 FLUORESCENCE MICROSCOPY IMAGE ANALYSES

The fluorescence microscopy images of unexposed tubulin and tubulin exposed to the Vielight LED presented in Section 4.1 were analyzed using the Image Processing Toolbox in MATLAB®. For every image presented in Figures 6 and 7, the RGB color profile was sampled over each of the pixels. Specifically, the `improfile` function was used, which obtains the RGB value (as brightness ranging from 0–255) for each pixel cross-section along a user-defined line segment. This was performed iteratively by placing horizontal line segments across the images at every pixel in the vertical (y) direction, and then averaging the values obtained (over the y-direction) for each set of control and exposed MT images presented. This provides the average RGB brightness value calculated at every pixel along the x-direction for each set of three images provided in Figures 6 and 7. The results obtained in the red band, for the experiments that used a high Taxol™ concentration of 20 μM, are shown in Figure S1. Additionally, a single mean brightness value was calculated in the red band for each image presented in Figures 6 and 7 by averaging the values over the horizontal pixels across the images as well. These mean red brightness values obtained for each individual image are provided in Table S1. For the tubulin samples with 20 μM Taxol™, the overall average red brightness values obtained for both sets of three control and exposed images were 124.1 ± 34.77 and 102.1 ± 37.83, respectively. This process was repeated for the images obtained in the experiments that used an intermediate Taxol™ concentration, yielding the curves shown in Figure S2. As expected, a greater difference was obtained between the overall mean red brightness values calculated for the control and Vielight-LED exposed MT images. In particular, the average value for the set of images of exposed MTs was only 95.4 ± 33.18.

Table S1. The average red brightness value of pixels calculated for each of the fluorescence microscopy images presented in Figures 6 and 7 of the main text.

Group	Image	Average Red Brightness
Control	Figures 6/7A	122.84 ± 33.51
	Figures 6/7B	131.24 ± 35.44
	Figures 6/7C	118.13 ± 35.32
Exposed (20 μM Taxol™)	Figure 6D	102.59 ± 36.08
	Figure 6E	99.17 ± 40.73
	Figure 6F	104.50 ± 36.51
Exposed (4 μM Taxol™)	Figure 7D	78.65 ± 31.15
	Figure 7E	133.80 ± 38.07
	Figure 7F	73.77 ± 29.71

2 SIGMOIDAL FITS

The tubulin turbidity datasets were analyzed using the nonlinear curve fitting tool available in the software ORIGINPRO®. To perform outlier removal, the turbidity curves obtained for the tubulin samples reconstituted with GTP exposed to the Vielight LED were filtered with a fast Fourier transform (FFT) filter with a 5 point window and a cut off frequency of 0.0033 Hz. The data were then fit to the Boltzmann model which consists of a sigmoidal curve governed by the following equation,

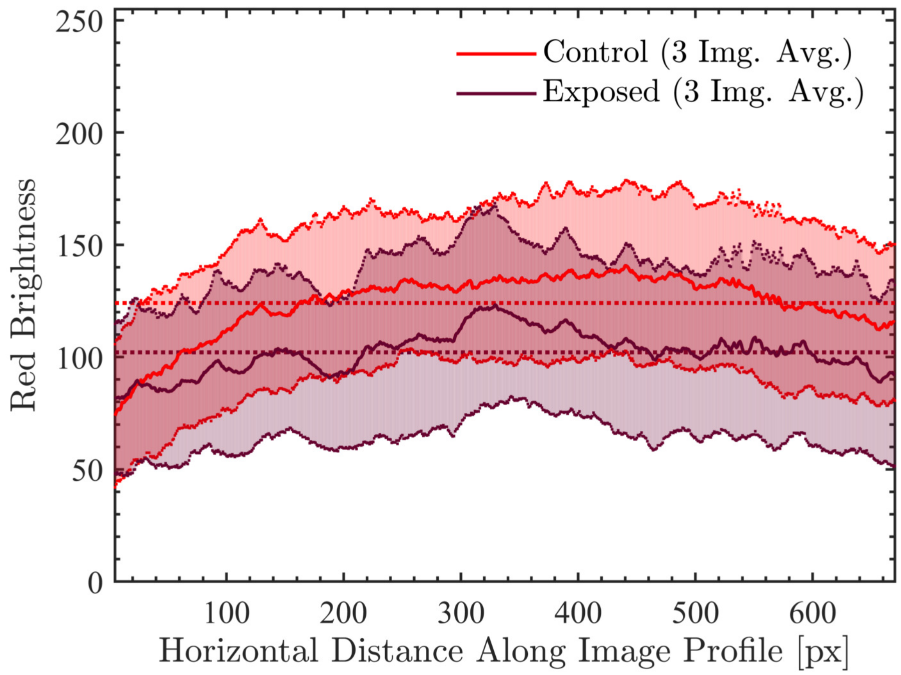

Figure S1. The average red brightness values of the horizontal pixels from each of the 3 control (solid red) and 3 Vielight-LED exposed (solid maroon) microtubule images obtained by fluorescence microscopy in the experiment that used a high Taxol™ concentration of 20 µM (presented in Figure 6 of the main article). The dotted lines overlayed represent the mean value of each curve, 124.1 for the control and 102.1 for the exposed.

$$y(t) = A_2 + \frac{A_1 - A_2}{1 + e^{\frac{t-t_0}{\tau}}}, \tag{S1}$$

where A_1 is the initial y value, A_2 is the maximum y value reached (plateau), τ is the time constant, and t_0 is the t value when 50% of the threshold is reached. These parameters are represented visually in Figure S3. Specifically, the corresponding input data were pre-selected and then the `NLFit` dialog box in ORIGINPRO® was used to select the Boltzmann function and apply the fits. The results of each sigmoidal fit obtained in this way are presented in Tables S2, S3, and S4 for the 22.7 µM tubulin, 45.5 µM tubulin, and 22.7 µM tubulin reconstituted with exposed GTP, respectively. The resulting fits are plotted alongside the original (average) curves for each corresponding dataset in Figures S4, S5, and S6.

3 ANALYSES OF MAXIMAL SLOPES & TENTH TIMES

In order to obtain the maximal slope values (i.e. the slope evaluated at the inflection point, t_0) associated with each turbidity curve presented, we first differentiate Equation S1 with respect to time. We find the following result for the first derivative of the Boltzmann sigmoidal equation,

$$y'(t) = \frac{(A_2 - A_1)\, e^{\frac{t-t_0}{\tau}}}{\tau \left(1 + e^{\frac{t-t_0}{\tau}}\right)^2}. \tag{S2}$$

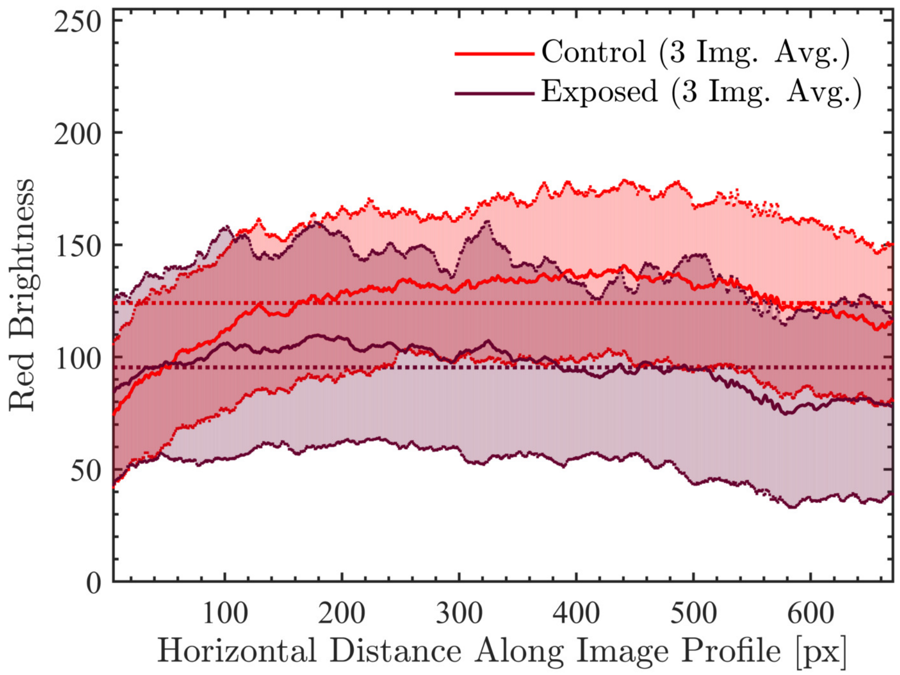

Figure S2. The average red brightness values of the horizontal pixels from each of the 3 control (solid red) and 3 Vielight-LED exposed (solid maroon) microtubule images obtained by fluorescence microscopy in the experiment that used an intermediate Taxol™ concentration of 4 µM (presented in Figure 7 of the main article). The dotted lines overlayed represent the mean value of each curve, 124.1 for the control and 95.4 for the exposed.

Table S2. The resulting parameter values obtained for sigmoidal fits applied to the 22.7 µM tubulin turbidity data.

	Sample (22.7 µM Tubulin)	
**Parameter**	**Control**	**Exposed**
A_1 [OD]	$(-3.41 \pm 0.25) \times 10^{-3}$	$(-1.81 \pm 0.23) \times 10^{-3}$
A_2 [OD]	$(8.734 \pm 0.036) \times 10^{-2}$	$(7.407 \pm 0.057) \times 10^{-2}$
t_0 [s]	1366.55 ± 3.71	1559.63 ± 6.05
τ [s]	272.94 ± 3.64	298.99 ± 5.33
Reduced χ^2	7.46×10^{-7}	7.13×10^{-7}
R^2 (COD)	0.99937	0.99897
Adj. R^2	0.99934	0.99893

Evaluating Equation S2 at the inflection point, t_0, yields the maximal value of the slope, $y'(t = t_0) = V_{\max} = \frac{(A_2 - A_1)}{4\tau}$. Standard error propagation is used to obtain the uncertainty in the maximal slope value,

$$\sigma_{V_{\max}} = |V_{\max}| \sqrt{\frac{(\sigma_{A_1})^2 + (\sigma_{A_2})^2}{(A_2 - A_1)^2} + \left(\frac{\sigma_\tau}{\tau}\right)^2}. \qquad \text{(S3)}$$

The tenth time associated with each turbidity curve was calculated following the approach of Bonfils et al. (Ref. [58] in the main text). This was done by directly calculating the value of time when the OD_{340} has reached 10% of its maximal value (obtained as A_2 from the previously described sigmoidal fits).

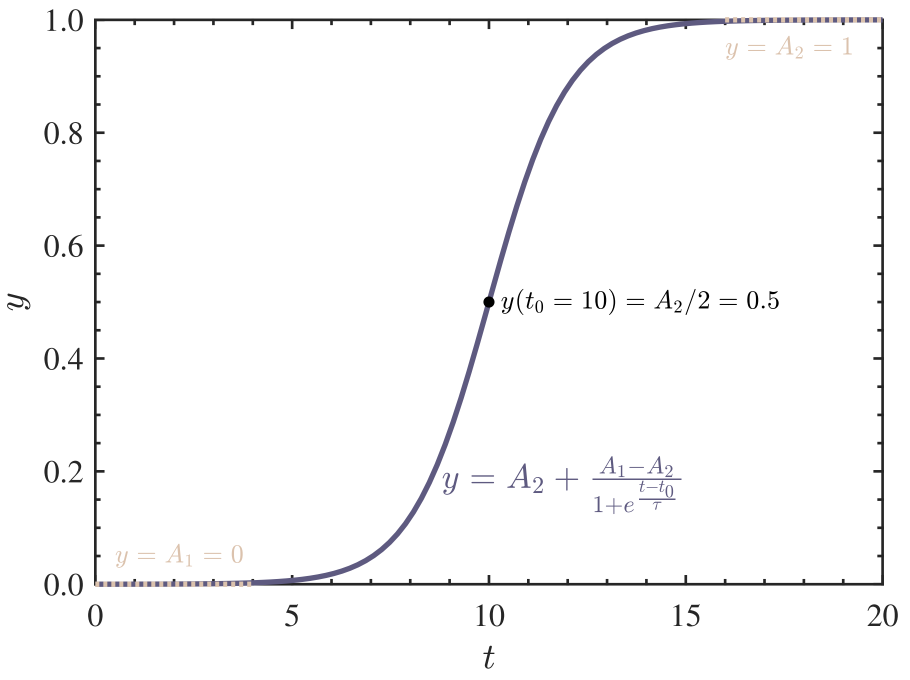

Figure S3. A visual representation of the parameters associated with the Boltzmann sigmoid function that was fit to the turbidity datasets.

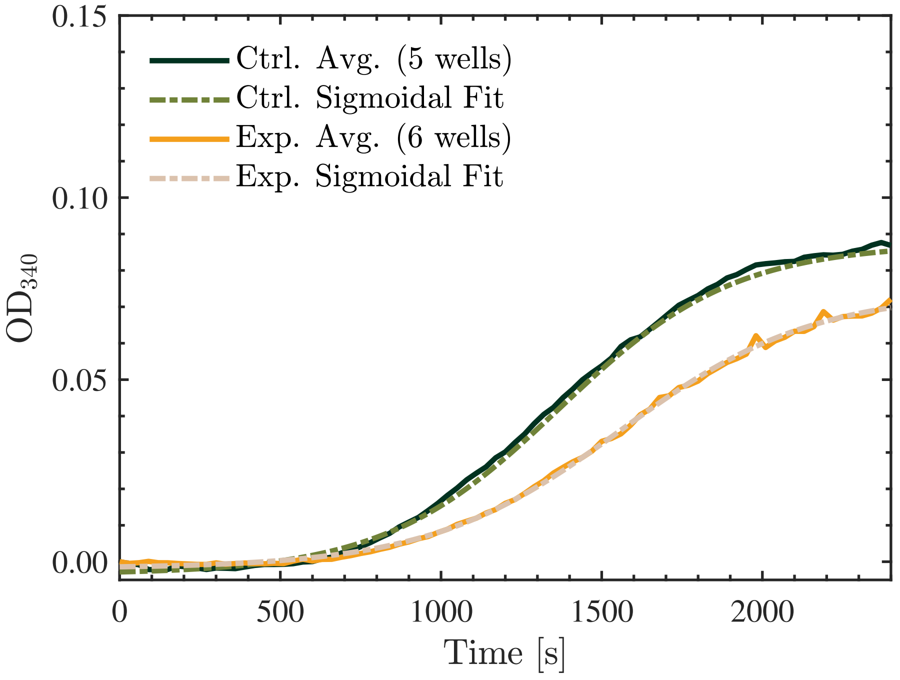

Figure S4. The resulting sigmoidal fits (dash-dotted lines) obtained for the 22.7 μM tubulin control (olive green) and exposed (beige) turbidity data overlayed against the original data (solid green and yellow lines).

A summary of all of these results can be found in Table S5.

Table S3. The resulting parameter values obtained for sigmoidal fits applied to the 45.5 μM tubulin turbidity data.

	**Sample (45.5 μM Tubulin)**	
**Parameter**	**Control**	**Exposed**
A_1 [OD]	$(-3.02 \pm 0.86) \times 10^{-3}$	$(-1.54 \pm 0.91) \times 10^{-3}$
A_2 [OD]	$(1.397 \pm 0.004) \times 10^{-1}$	$(1.812 \pm 0.004) \times 10^{-1}$
t_0 [s]	698.60 ± 3.53	571.59 ± 2.28
τ [s]	121.64 ± 3.04	82.52 ± 1.96
Reduced χ^2	6.75×10^{-6}	7.91×10^{-6}
R^2 (COD)	0.998	0.99849
Adj. R^2	0.99793	0.99843

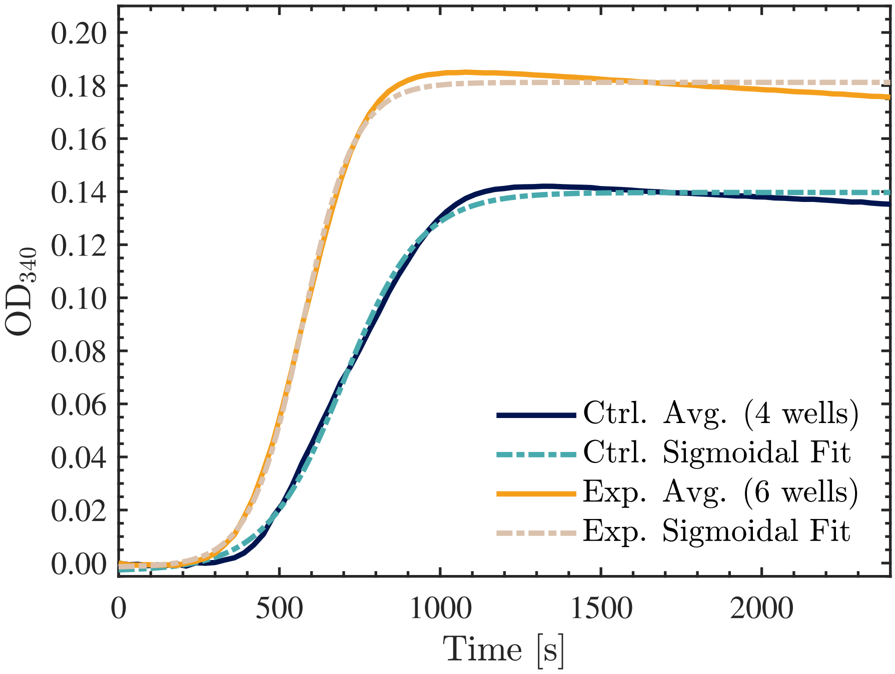

Figure S5. The resulting sigmoidal fits (dash-dotted lines) obtained for the 45.5 μM tubulin control (light blue) and exposed (beige) turbidity data overlayed against the original data (solid navy and yellow lines).

Table S4. The resulting parameter values obtained for sigmoidal fits applied to the turbidity data from the experiment with 22.7 μM tubulin reconstituted with GTP exposed to the Vielight LED.

	**Sample (22.7 μM Tubulin)**	
**Parameter**	**Control**	**Exposed GTP**
A_1 [OD]	$(-3.28 \pm 0.22) \times 10^{-3}$	$(-2.16 \pm 0.27) \times 10^{-3}$
A_2 [OD]	$(9.058 \pm 0.025) \times 10^{-2}$	$(1.070 \pm 0.003) \times 10^{-1}$
t_0 [s]	1264.78 ± 2.71	1322.99 ± 3.03
τ [s]	258.94 ± 2.68	224.72 ± 2.85
Reduced χ^2	5.26×10^{-7}	1.19×10^{-6}
R^2 (COD)	0.99961	0.99939
Adj. R^2	0.99959	0.99937

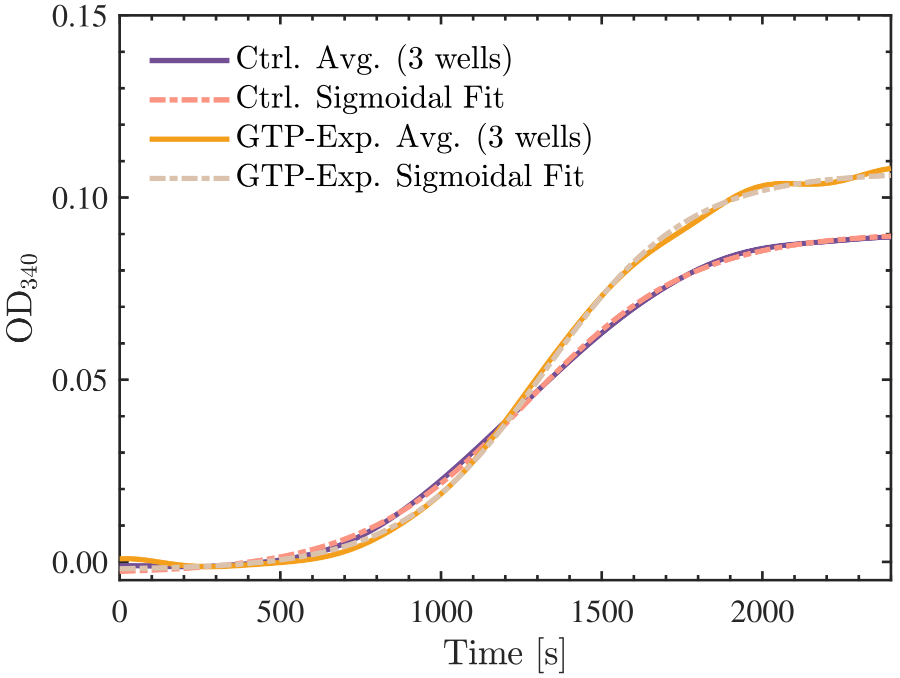

Figure S6. The resulting sigmoidal fits (dash-dotted lines) obtained for the Vielight LED-exposed GTP experiment, for the 22.7 µM tubulin control (peach) and exposed (beige) turbidity data overlayed against the original data (solid purple and yellow lines).

Table S5. The calculated maximal slopes and tenth times for each set of unexposed and Vielight-LED exposed tubulin turbidity data: 22.7 µM tubulin, 45.5 µM tubulin, and 22.7 µM tubulin reconstituted with GTP exposed to the Vielight LED.

**Scenario**	**Sample**	**Maximal Slope** (V_{max}) [mOD/min]	**Tenth Time** ($t_{1/10}$) [s]
22.7 µM tubulin	Control	5.0 ± 0.1	870
	Exposed	3.8 ± 0.1	990
45.5 µM tubulin	Control	17.6 ± 0.5	480
	Exposed	33.2 ± 0.8	420
22.7 µM tubulin & exposed GTP	Control	5.4 ± 0.1	810
	Exposed	7.3 ± 0.1	900
